# Closing the Organofluorine Mass Balance in Marine Mammals Using Suspect Screening and Machine Learning-Based Quantification

**DOI:** 10.1021/acs.est.3c07220

**Published:** 2024-01-25

**Authors:** Mélanie Z. Lauria, Helen Sepman, Thomas Ledbetter, Merle Plassmann, Anna M. Roos, Malene Simon, Jonathan P. Benskin, Anneli Kruve

**Affiliations:** † Department of Environmental Science, Stockholm University, Svante Arrhenius Väg 8, 10691 Stockholm, Sweden; ‡ Department of Materials and Environmental Chemistry, Stockholm University, Svante Arrhenius Väg 16, 106 91 Stockholm, Sweden; § Department of Environmental Research and Monitoring, Swedish Museum of Natural History, 104 05 Stockholm, Sweden; ∥ Greenland Climate Research Centre, Greenland Institute of Natural Resources, 3900 Nuuk, Greenland

**Keywords:** Combustion ion chromatography, high resolution mass spectrometry, suspect screening, ionization efficiency-based quantification, dolphins, cetaceans

## Abstract

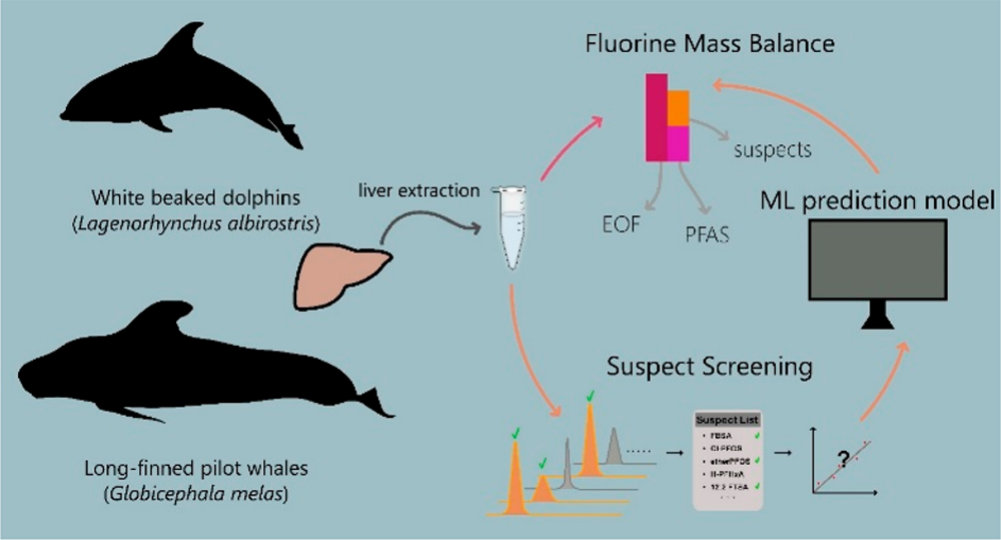

High-resolution mass spectrometry (HRMS)-based suspect and nontarget screening has identified a growing number of novel per- and polyfluoroalkyl substances (PFASs) in the environment. However, without analytical standards, the fraction of overall PFAS exposure accounted for by these suspects remains ambiguous. Fortunately, recent developments in ionization efficiency (*IE*) prediction using machine learning offer the possibility to quantify suspects lacking analytical standards. In the present work, a gradient boosted tree-based model for predicting log *IE* in negative mode was trained and then validated using 33 PFAS standards. The root-mean-square errors were 0.79 (for the entire test set) and 0.29 (for the 7 PFASs in the test set) log *IE* units. Thereafter, the model was applied to samples of liver from pilot whales (n = 5; East Greenland) and white beaked dolphins (n = 5, West Greenland; n = 3, Sweden) which contained a significant fraction (up to 70%) of unidentified organofluorine and 35 unquantified suspect PFASs (confidence level 2–4). *IE*-based quantification reduced the fraction of unidentified extractable organofluorine to 0–27%, demonstrating the utility of the method for closing the fluorine mass balance in the absence of analytical standards.

## Abstract 2

A machine-learning log *IE* prediction model was trained and validated using a suite of analytical PFAS standards; thereafter, the model was applied to quantify PFAS suspects observed in marine mammal livers.

## Introduction

Per- and polyfluoroalkyl substances (PFASs) are defined as chemicals containing at least one fully fluorinated methyl (−CF_3_) or methylene (−CF_2_−) group without hydrogen, chlorine, bromine, or iodine atoms.^1^ According to PubChem, > 7 million substances fall under this definition, although the commercial relevance of all of these chemicals remains unclear.^2^ PFASs are widely used in industrial or consumer applications, with over 200 different uses for more than 1400 individual substances.^3^ While many PFASs are hazardous^4^ and/or prone to large-range transport,^5,6^ persistence is considered a common property of the entire class and the principal cause for concern.^7^

Due to their large number and diversity of structures, PFAS contamination in the environment is highly complex. Recent studies involving organofluorine mass balance and suspect screening have determined that exposure to PFASs in marine mammals using targeted analytical methods can be considerably underestimated.^8−11^ Given their position at the top of the marine food web, marine mammals are used globally for monitoring persistent and bioaccumulative pollutants in the marine environment.^12,13^ Moreover, since they partly share their diet with humans (e.g., fish, crustaceans, cephalopods) and are regularly consumed by Arctic communities , marine mammals can be used as early indicators of emerging contaminants relevant for human exposure through diet and other environmental exposure media.^14^

The large number of suspect PFASs detected by liquid chromatography high-resolution mass spectrometry (LC-HRMS) and relatively few analytical standards for quantification has provided considerable impetus for development of alternative quantitative approaches. Traditionally, analytical standards are required for quantification in LC-HRMS in order to compensate for the wide range of ionization efficiency (*IE*) among chemicals, *i*.*e*., the number of ions produced in the ionization source from a given concentration of analyte. *IE* is highly variable even within one group of structurally similar chemicals.^15^ For example, when measured under the same conditions and concentrations, 2,4-dinitrophenol can produce a 380-fold higher signal compared to 2-nitrophenol.^16^ For PFAS analysis, it is common to quantify substances that lack analytical standards using a homologue that is close in carbon chain length. For example, the concentration of perfluoroheptanesulfonic acid (PFHpS) has been estimated based on the calibration curve of perfluorohexanesulfonic acid (PFHxS).^17^ In recent years, machine learning-based models that incorporate molecular and eluent descriptors have shown considerable potential for predicting *IE*. Compared to the homologue quantification approach, machine learning models have a clear advantage: they can be used for any carbon number as well as for chemicals for which no analytical standards are available in the homologue series. The application of such tools depends on the chemical space covered by the data used for training these models; therefore, they need to be retrained and revalidated to expand the application domain for new chemical classes.

The overarching goal of this study was to develop a quantification tool to determine the organofluorine mass balance in environmental samples in the absence of analytical standards. To achieve this goal, we increased the scope of a previously published quantification model by including 33 additional PFASs in model training. Thereafter, the model was applied to quantify PFASs lacking an analytical standard detected via suspect screening in marine mammal livers, thereby reducing the fraction of unidentified extractable organofluorine (UEOF) in these samples. To the best of our knowledge, this is the first time a machine learning-based quantitative approach has been used to help close the organofluorine mass balance in environmental samples.

## Materials and Methods

### Sample Collection

Liver samples were collected from 5 long-finned pilot whales (*Globicephala melas*) from East Greenland and 5 white-beaked dolphins (*Lagenorhynchus albirostris*) from West Greenland in 2018 and 3 white-beaked dolphins from the West coast of Sweden between 2007 and 2009. All animals from Greenland were obtained as part of subsistence hunting, while individuals from Sweden were found stranded. Samples will be referred to in relation to their sampling location, but it is important to note that these animals can travel long distances during their lives. All samples were stored at −20 °C until analysis. Portions of liver from one pilot whale and one dolphin were analyzed in triplicate, and their relative standard deviation (RSD) was used for the other individuals of the same species. Further details on sample collection, including shipping permits from the Convention on International Trade in Endangered Species of Wild Fauna and Flora (CITES), can be found in Table S1.

### Chemicals and Reagents

Both native- and isotopically labeled internal standards (ISs) included in the targeted analysis were purchased from Wellington Laboratories (Guelph, Canada) and are listed in Table S2. Among the 33 target PFASs were 11 perfluoroalkyl carboxylic acids (PFCAs, C_5–14,16_), 4 perfluoroalkyl sulfonic acids (PFSAs, C_4,6,8,10_), 3 fluorotelomer carboxylic acids (3:3, 5:3, and 7:3 FTCAs), 3 fluorotelomer sulfonic acids (4:2, 6:2, and 8:2 FTSAs), 3 (*N*-alkyl) perfluoroalkane sulfonamides (FASAs; FOSA, MeFOSA, and EtFOSA), 3 (*N*-alkyl) perfluoroalkane sulfonamidoacetic acids (FASAAs; FOSAA, MeFOSAA, and EtFOSAA), 3 polyfluoroalkyl phosphoric acid diesters (6:2, 6:2/8:2, and 8:2 diPAP), 2 chlorinated perfluorinated ether sulfonates (Cl-PFESAs; 9Cl-PF3ONS [also known as 6:2 Cl-PFESA] and 11Cl-PF3OUdS [also known as 8:2 Cl-PFESA]), and 4,8-dioxa-3H-perfluorononanoic acid (ADONA). Other chemicals and reagents are provided in the SI.

### Sample Preparation for Organofluorine Mass Balance

A schematic overview of the organofluorine mass balance approach used in this study can be found in Figure S1. Liver samples were thawed at room temperature, and subsampling was carried out with a stainless-steel knife precleaned with methanol. Extraction was carried out using a previously published method,^9^ which is described in detail in the SI. Briefly, ∼0.5 g of liver was extracted twice with acetonitrile and bead blending followed by a dispersive carbon cleanup. The final extract was split into two aliquots of 250 μL each. The first aliquot (destined for HRMS-based target and suspect screening) was transferred to another Eppendorf tube and fortified with ISs and buffer. The second aliquot, destined for extractable organofluorine (EOF) analysis by Combustion Ion Chromatography (CIC), was transferred to a new Eppendorf tube. All extracts were stored at −20 °C. Upon analysis, the extracts were adjusted to room temperature, vortexed, and transferred to LC vials.

### Instrumental Analysis

#### EOF Analysis

CIC measurements were carried out with a Thermo-Mitsubishi CIC and based on previously described methods (see Table S3 and the Supporting Information section on *EOF analysis* for details).^9,18^ Briefly, samples were combusted for 5 min at 1100 °C under a flow of gases, which were absorbed in Milli-Q water. A portion of this water was injected onto an IC for determination of fluoride. The mean fluoride concentration from procedural blanks was subtracted from the samples before quantification and the limit of quantification (LOQ; 29.3 ng F/g) was calculated using 3 times the standard deviation of fluoride in the procedural blanks (n = 3 for each batch).

#### Target Analysis and Suspect Screening

Target and suspect screening analyses were carried out simultaneously using a previously validated method,^9,19^ described in detail in the SI. Briefly, sample extracts were injected onto a Dionex ultra high-performance liquid chromatograph (UHPLC) equipped with a C18 column and coupled to a Q-Exactive Orbitrap^TM^ mass spectrometer via an electrospray ionization (ESI) source. The instrument was operated in negative ionization, full-scan (200–1800 *m*/*z*, resolution 120 000) data-dependent MS^2^ acquisition (DDA, resolution 15 000) mode, based on an inclusion list of molecular ions for 324 known PFASs. The list was established from prior publications reporting novel PFASs and monofluorinated substances in marine mammals, birds, and fish and is provided as Table S4.^9,10,20−23^

Quantification of targets was carried out using Thermo Scientific TraceFinder^TM^ Software version 4.1. Relative response factors were used for quantification in the linear range with 1/x weighting. A second data processing method was created in TraceFinder^TM^ for suspects, and those with peak heights > 10 000 cps were considered significant. Using the Thermo Excalibur Qual browser, each detected suspect’s MS^2^ spectra were checked for fragments which could confirm their identity by comparing with literature information. Confidence Levels (CLs) were assigned to suspects according to the scale proposed by Schymanski et al. (see overview in the SI).^24^

#### Quality Control

A suite of QC samples was used to ensure the accuracy and precision of EOF and target PFAS data. This included: spiked liver (with native nonisotopically labeled PFASs and with inorganic fluorine, both n = 3), procedural blanks (n = 3), and analysis of certified reference materials (fluorine in clay, n = 3). Spiked liver and procedural blanks were analyzed in the same manner as other samples, and background subtraction (either using unspiked liver for spiked QC samples and procedural blanks for liver samples) was performed prior to calculations. Results of these experiments, which are described in detail in the SI, demonstrated acceptable accuracy and precision across all analyses.

### Developing Ionization Efficiency Prediction Model for Quantification

#### Data Preparation

Calibration curves based on molar concentration vs peak area (weighting: 1/concentration; including intercept) were constructed for 33 target PFASs using experimental data of the calibration mix from the targeted analysis of the samples. To account for all ions formed through the same ionization mechanism, peak areas from [M-H]^−^ ions and their in-source fragments were summed to provide the signal for each compound in the calibration mix. The in-source fragments were identified by a careful manual evaluation of the full-scan spectra. Additionally, the detected monoisotopic mass peak area was corrected for uncounted isotope peaks by calculating the isotope distribution from the molecular formula. Absolute response factors (*RF*s) were obtained as the slopes of the calibration graph for each compound. The linearity was checked based on relative residuals and considered acceptable if the relative residual with highest absolute value was ≤20%.

The *RF*s were used to expand a previously combined data set by Liigand et al.^16^ For this, the *RF*s of 33 target PFASs were converted to relative *IE* values by anchoring the data using the response factor of PFOS that was present in both data sets. To calculate the *IE* value for any of the 33 remaining PFASs (*IE*_M_), the following equation was used
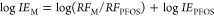
1where *RF*_M_ is the experimental response factor of the respective PFAS, *RF*_PFOS_ is the experimental response factor of PFOS measured in the same sequence as the respective PFAS, and *IE*_PFOS_ is the *IE* value of PFOS from the data set of Liigand et al.^16^ Experimental conditions such as organic modifier percentage during elution, pH, and additives used in the measurements affect the *IE* of a chemical. In the Liigand et al. data set, experimental conditions were as follows: pH of 7.8 with an ammonium acetate buffer and an organic modifier content of 80% acetonitrile at time of elution, resulting in the relative ionization efficiency value of log *IE*_PFOS_ = 2.59 log-units. For the experimental response factor of PFOS obtained in the target analysis of this study, the conditions were as follows: organic modifier content 52% acetonitrile at time of elution and pH of 7.0 with 1 mM ammonium acetate buffer resulting in *RF*_PFOS_ = 11.56 log-units. These conditions were considered sufficiently similar to assume differences in ionization efficiency as insignificant. Additionally, all *IE* values in this study are relative.

#### Modeling

##### Original Data Set for Modeling

The first model was trained using 100 unique chemicals measured in negative mode under different experimental conditions such as organic modifier percentage, mobile phase pH, and additive type, resulting in 1286 data points.^16^ Out of the 100 chemicals, 19 were fluorinated: PFOS, perfluoro-*tert*-butanol, 11 aromatic PFASs, and 6 aromatic organofluorine compounds that do not fall under the PFAS definition.

##### Data Set with Additional PFASs

The ESI negative mode prediction model developed in this study was trained on a set of 132 unique chemicals (including 33 PFASs measured for this study and 13 that were present in the original data set compiled by Liigand et al.)^16^ and altogether 1319 data points measured under different eluent conditions.

##### Descriptors for Modeling

For training the model, the chemical structures were translated into numerical molecular descriptors. Pharmaceutical Data Exploration Laboratory (PaDEL)^25^ molecular descriptors have shown good predicting power for *IE*s^16,26,27^ and were therefore used in the present work. These molecular descriptors include both structural information such as atom and bond count descriptors as well as more complex descriptors such as Topological Distance Matrix and Electrotopological State Atom Type descriptors, which were calculated off-line from the Simplified Molecular-Input Line-Entry System (SMILES) notation of a chemical with code provided by the Chemical Development Kit. In addition to PaDEL descriptors, five eluent descriptors were used (aqueous pH, polarity index, viscosity, surface tension, and presence of NH_4_^+^) to account for the effect of the mobile phase composition.

Prior to modeling, the data set was cleaned to remove correlated or noninformative columns to avoid overtraining (details in the SI). For data processing, feature calculations and modeling, R version 4.1.1 was used. The code, data, and models are provided on GitHub page https://github.com/kruvelab/PFAS_quantification_model.

##### Model Performance Evaluation Parameters

The model’s prediction power was assessed based on the root-mean-square error (RMSE) of the training and test sets, as well as calculating the fold prediction error for *IE* and concentration predictions using the following equation:
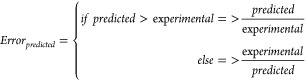
2The mean, median, and geometric mean of fold prediction errors were used to compare model performances.

##### Ionization Efficiency Prediction Model Training

To evaluate the model’s performance after adding PFASs to the training of the model, the data was divided into training (80%) and test (20%) sets with stratified sampling so that 80% of PFASs would be in the training set. The regression model for log *IE* predictions was trained using the extreme gradient boosting tree (*xgbTree*) algorithm. The hyperparameters were optimized with the bootstrap resampling method (*boot*) using cross-validation with five sets. As chemicals in the original data set had multiple log *IE* values that were measured under different experimental conditions, all data points were used in training and testing the model; however, cross-validation sets were generated using chemical names with the *groupKFold()* function to avoid the same chemical ending up in both training and test subsets. For the final model, the optimized hyperparameters were used to train a model based on all data in the combined data set. More detailed model analysis and the 10 most impactful descriptors in the final model can be found in Table S5.

##### Leave-one-out Modeling Approach for Quantification

To compare the performance of prediction for individual PFASs between the model trained on the original data set and the model trained with the additional PFAS data, the leave-one-out approach was used. For this, 33 models were trained - one for each of the added PFASs. In these models, the individual PFAS being evaluated was left out from the training data set, while all other data points were used for modeling. Then the model was used to predict the log *IE* for the PFAS that was left out, and the results were compared to the experimental log *IE* of the respective PFAS.

##### Homologue Series Approach for Quantification

One approach for quantifying a PFAS without an analytical standard is to use another PFAS within the same homologue series (i.e., with a different carbon chain length). Therefore, we compared the performance of quantification using predicted log *IE* values versus using the homologue series approach. For this, we determined all close homologues present in our data set and used the response factors of these chemicals for quantification. In total, there were 10 homologues that had a carbon chain difference of −CF_2_– and 26 with a difference of −C_2_F_4_–. In case smaller and larger homologues were present, both were used for quantification, and the results were averaged. The overview of all homologues and comparison between model and −C_2_F_4_– homologue quantification can be found in Tables S6–S7 and Figure S2.

##### Calculating Concentration Using Predicted Ionization Efficiencies

**Figure 1:**
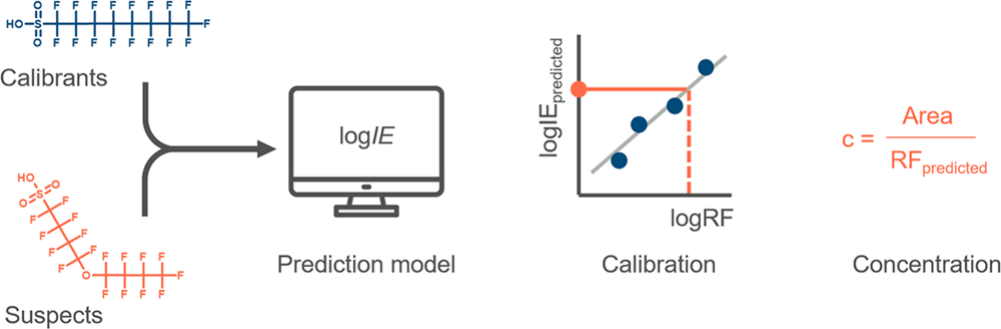
Workflow for obtaining predicted concentrations for suspects detected in liver samples using predicted log *IE* values. The 33 target PFASs were used as calibrants for converting the predicted log *IE* of the suspect chemicals to the predicted response factor, which was used in concentration estimations.

For quantification approaches using a homologue (i.e., homologue series approach), the *RF* of a structurally similar PFAS is assumed to be insignificantly different from the detected chemical, and therefore, the *RF* of the homologue is used instead to calculate the concentration:
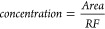
3In the case of the *IE*-prediction model, the predicted log *IE* indicates how the *RF* of a respective chemical relates to other chemicals and is instrument independent. However, *RF*s are instrument- and lab-specific, meaning that the magnitude of *RF* depends on the source design, instrument vendor, and to some extent even on the software used for data integration (units, peak picking, integration, etc.). Therefore, predicted *IE* needs to be converted to measurement-specific *RF* before quantification. For this, the 33 target PFASs could be used as calibrants because they were measured together with the suspects we aimed to quantify. For the target PFAS, a calibration graph between measurement-specific response factors and predicted *IE*s was constructed. Using this calibration graph, the log *IE* was predicted for suspect chemicals detected in liver samples, converted into predicted *RF*, and used to calculate the concentration (Figure 1).

##### Statistical Significance

Due to non-normal and asymmetrical distribution of the fold prediction errors, the statistical significance between errors arising from different quantification approaches was tested with paired two-sided Wilcoxon signed rank tests using a level of significance of α = 0.05.

### Fluorine Mass Balance Calculations

To facilitate comparisons with EOF measurements, PFASs quantified using analytical standards or the *IE*-prediction model were converted to fluorine equivalent concentrations (i.e., *C*_F_PFAS_ in ng F/g):
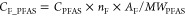
4*C*_PFAS_ is a PFAS concentration in ng/g, *n*_F_ is the number of fluorine atoms on the compound, *A*_F_ is the average atomic weight of one fluorine atom in g/mol, and *MW*_PFAS_ is its molecular weight. The sum of concentrations of target PFASs (i.e., ∑PFAS) or suspects (i.e., ∑Suspects) was obtained by summing *C*_F_PFAS_ values of a sample for individual targets or suspects, respectively.

## Results and Discussion

### Targeted Analysis and Organofluorine Mass Balance

A total of 16 out of 33 target PFASs were quantified in one or more samples (Table S8). Concentrations are displayed here with up to 2 significant figures, and raw concentrations can be found in the SI (Tables S8 and S9). The highest average ∑PFAS concentrations were observed in dolphins from Sweden (620 ± 220 ng/g wet weight [ww]; 410 ± 150 ng F/g ww), followed by East Greenland pilot whale (220 ± 64 ng/g ww; 150 ± 43 ng F/g ww) and West Greenland dolphin (78 ± 14 ng/g ww; 53 ± 10 ng F/g ww). Across all Greenlandic samples, PFAS profiles were dominated by PFOS (up to 94 ng/g), PFUnDA (up to 66 ng/g), and PFTriDA (up to 58 ng/g), which collectively accounted for ∼72% of ∑PFAS (and ∼90% when including all PFCAs), consistent with prior observations in cetaceans from the Nordic environment.^9,28^ Swedish dolphins also displayed high PFOS concentrations (up to 300 ng/g) but with significant contributions from 7:3 FTCA (up to 220 ng/g) and FOSA (up to 250 ng/g), collectively accounting for ∼80% of ∑PFAS (Figure S3). 7:3 FTCA is a stable transformation product of fluorotelomer alcohols^29−31^ and has been previously measured in marine mammals and seabirds globally,^9−11,20,32−34^ while FOSA has been previously reported at elevated concentrations in cetaceans^9,10,20,35,36^ due to the limited capacity of these species to biotransform FOSA into PFOS.^37,38^

**Figure 2:**
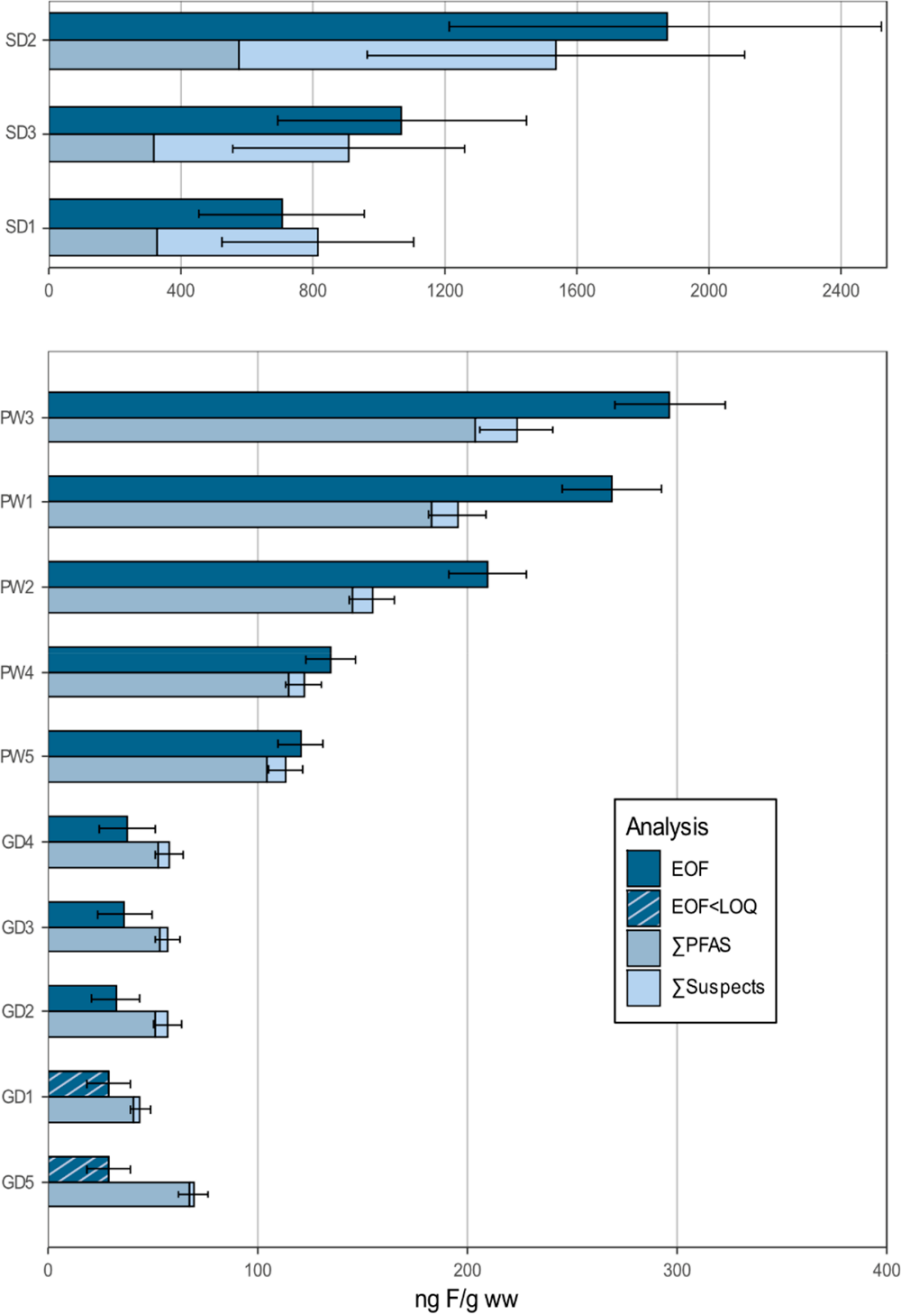
Fluorine mass balance for dolphins from Sweden (SD), pilot whales from East Greenland (PW), and dolphins from West Greenland (GD) in decreasing order of EOF concentrations from top to bottom. Model error is not plotted here, error bars are based on relative standard deviation observed for triplicate measurements of GD3 (for dolphins) and PW-3 (for pilot whales), and the standard deviations of ∑PFAS and ∑Suspects are combined. GD1 and -5 have EOF < LOQ; therefore, the LOQ value (29.3) was plotted here.

EOF concentrations were also highest in dolphins from Sweden (1200 ± 600 ng F/g ww) followed by pilot whales from East Greenland (210 ± 78 ng F/g ww) and dolphins from West Greenland (33 ± 4 ng F/g ww; Figure 2, Table S9). The significantly higher EOF concentrations in Swedish dolphins might be due to proximity to more industrialized coastal regions, but considering differences in sampling time periods (the Swedish samples are ∼10 years older than the Greenlandic samples), geographical comparisons should be interpreted cautiously.

A comparison of target PFASs to EOF concentrations revealed a significant gap in the fluorine mass balance in Swedish dolphins (53–70% UEOF), while long-finned pilot whales from East Greenland displayed a modest fraction of UEOF (13–32%) and dolphins from West Greenland had a closed fluorine mass balance. Similar observations have been made previously in marine mammals from the Nordic and Baltic environments. For example, Spaan et al. observed largely closed fluorine mass balances in marine mammals from Sweden and Greenland, with the exception of some apex predators (polar bears and killer whales).^9^ Similarly, Kärrman et al. reported a white beaked dolphin from East Greenland (2016) with EOF below LOQ (158 ng F/g) and only one out of five studied pilot whales from the Faroe Islands (sampled 2017) with EOF above the LOQ (42 ng F/g) and a significant quantity of UEOF (80%).^28^ Additionally, a pooled sample of 6 white beaked dolphins from East Greenland (2016) had a concentration of ∼250 ng F/g,^28^ similar to pilot whales in the present study from the same region. Overall, EOF and fluorine mass balances were similar to those reported previously.

### Suspect Screening

A total of 35 suspects from 11 classes were detected between CLs 2 to 4. A summary of targets and suspects observed in this study can be found in Table 1, along with details on their *m*/*z*, formula, retention times, assigned CL, and observed fragments ions. Of the 35 suspects, 18 were part of a homologue series for which at least one homologue was included as a target (i.e., PFCAs, PFSAs, n:3 FTCAs, n:2 FTSAs, and FASAs). Three additional homologue series (encompassing 14 suspects), for which no homologue was included as part of the targeted analysis, were also discovered: chlorine substituted perfluorocarboxylic acids (C_9_ to C_14_), ether-PFSAs (C_6_ to C_8_), and a homologue series with formula C_*n*_F_2–9_H_9_NO_4_SH (C_12_ to C_16_) but unknown structure. The 3 remaining suspects were not part of a homologue series. Suspects are discussed by class in more detail below.

**Table 1:**
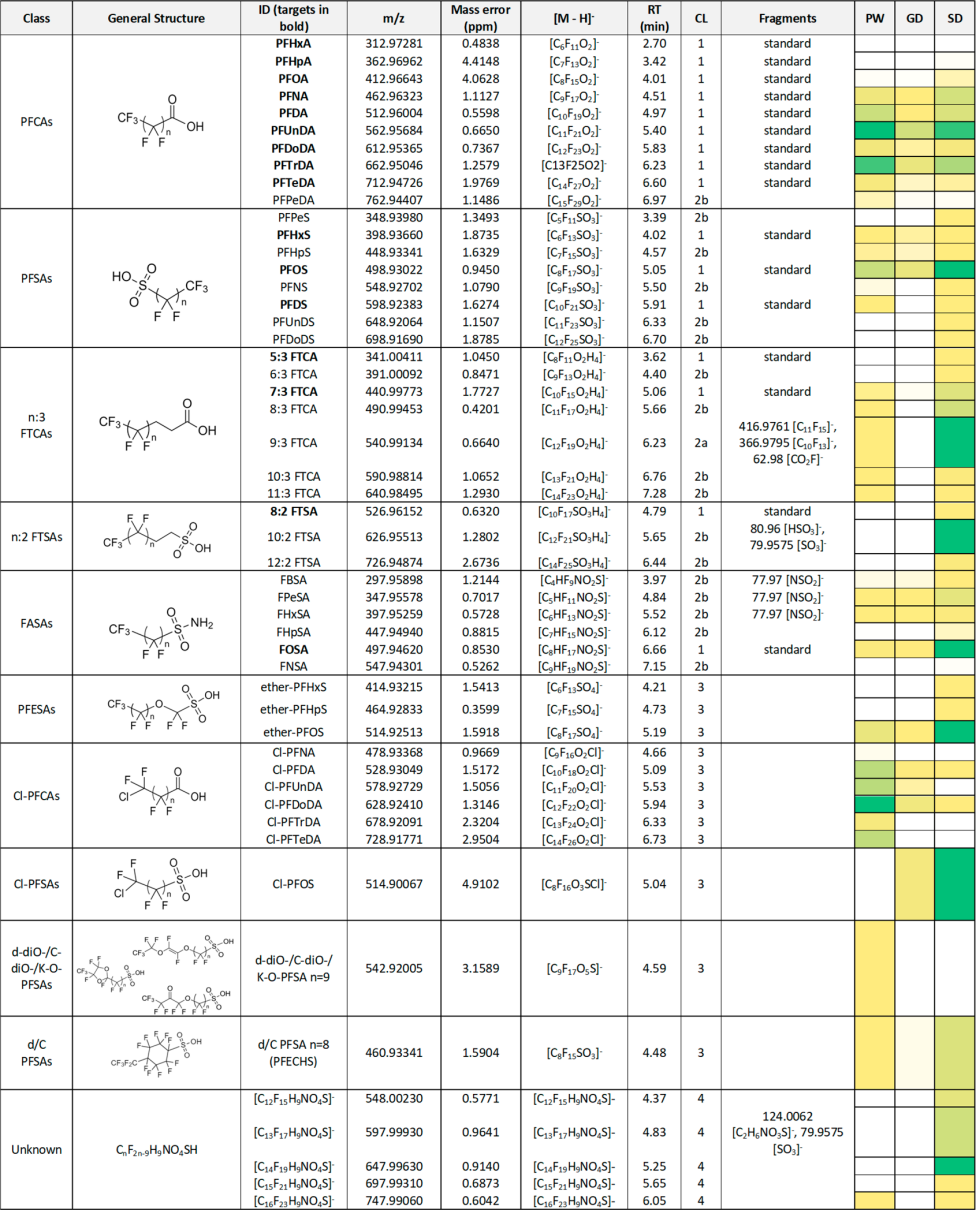
Heatmap of PFASs Identified by Target (in Bold) and Suspect Screening in Pilot Whales (PW, n = 5), West Greenland’s Dolphins (GD, n = 5), and Swedish Dolphins (SD, n = 3)^a^

a White represents nondetect and dark green represents the highest detect among sample groups. A different heatmap was created for each homologue series, except for the three compounds not part of a series, for which a joint heatmap was created. RT = retention time and CL= confidence level.

#### Classes 1 and 2: Perfluoroalkyl Acids

An additional PFCA (perfluoropentadecanoic acid, PFPeA) and five additional PFSAs were identified and assigned CL 2b, given MS^2^ data were unavailable, but RTs fitted the homologue series (Figures S4 and S5).

#### Classes 3 and 4: Fluorotelomer n:3 Carboxylic and n:2 Sulfonic Acids (n:3 FTCAs and n:2 FTSAs)

Five FTCAs in addition to the targets 5:3 and 7:3 FTCA were identified, of which 9:3 was assigned CL 2a from its MS^2^ matching the literature, and the others were assigned level 2b (Figures S6 and S7). Two FTSAs in addition to 8:2 FTSA were identified at level 2b: 10:2 and 12:2 FTSA, no MS^2^ was collected for 10:2 FTSA, while 12:2 FTSA showed [HSO_3_]^−^ and [SO_3_]^−^ fragments (Figures S8 and S9).

#### Class 5: Perfluoroalkane Sulfonamides (FASAs)

Five FASAs in addition to the targeted FOSA were identified at level 2b given the increasing RTs with an increasing chain length (Figure S10). Perfluorobutane, -pentane, and -hexane sulfonamide (FBSA, FPeSA, and FHxSA) MS^2^ spectra had the characteristic [NSO_2_]^−^ fragment (Figure S11), while MS^2^ was not triggered for perfluoroheptane and -nonane sulfonamide (FHpSA and FNSA).

#### Class 6: Chlorine Substituted PFCAs (Cl-PFCAs)

Six Cl-PFCAs (C_9_ to C_14_) were identified at level 3 since RTs increased with increasing chain length, but no MS^2^ data were acquired (Figure S12); therefore, the position of the chlorine substitution is not known, and only a probable structure is assigned. The presence of chlorine substitution is confirmed by the observation of the [^37^ClC_*n*_F_2*n*–2_O_2_]^−^ isotopologues, with approximately 30% relative abundance of [^35^ClC_*n*_F_2*n*–2_O_2_]^−^, for all homologues in the series (an example is shown in Figure S13).

#### Class 7: Ether PFSAs (PFESAs)

Ether-PFOS was found in samples of Swedish dolphins and Greenlandic pilot whales as well as in one Greenlandic dolphin; ether-PFHxS and ether-PFHpS were found in one sample of Swedish dolphins with increasing RT with increasing chain-length, but no MS^2^ data was obtained for this homologue series (Figure S14).

#### Classes 8 to 10

Three additional suspects, not part of a homologue series, were also discovered with CL 3: chlorine substituted perfluorooctanesulfonic acid (Cl-PFOS, from the Cl-PFSAs class), double bond-diether/cyclic-diether/ketone-ether perfluorononanoic sulfonic acid (d-diO/C-diO/K–O PFSA n = 9), and double bond/cyclic PFOS (d/C PFSA n = 8 or PFECHS).

#### Class 11: C_*n*_F_*2n*–9_H_9_NO_4_SH

An unknown homologue series, previously reported in marine mammals^9^ and in white-tailed sea eagle eggs,^34^ was also observed here, with molecular formula C_*n*_F_2–9_H_9_NO_4_SH (*n* = 12 to 16). It was not possible to assign a probable structure to this class (Figures S15 and S16).^9^

With the exception of the chlorine substituted substances and d-diO/C-diO/K–O PFSA n = 9, most of the aforementioned suspects were previously identified in various marine mammals from Greenland and Sweden by Spaan et al.^9^ Cl-PFCAs have been previously detected in wastewater from fluorochemical manufacturing parks,^39,40^ fish from the Yangtze river,^41^ and eggs of white-tailed sea eagle.^34^ Cl-PFSAs have also been previously identified in wastewater from a fluorochemical manufacturing park^40^ and in firefighters,^42^ and Cl-PFOS specifically has been identified in samples of marine mammals from the south China sea.^10^ D-diO/C-diO/K–O PFSA n = 9 has been previously identified in fish samples from Tangxun Lake, China.^41^

### Ionization Efficiency Model Development and Validation

**Figure 3:**
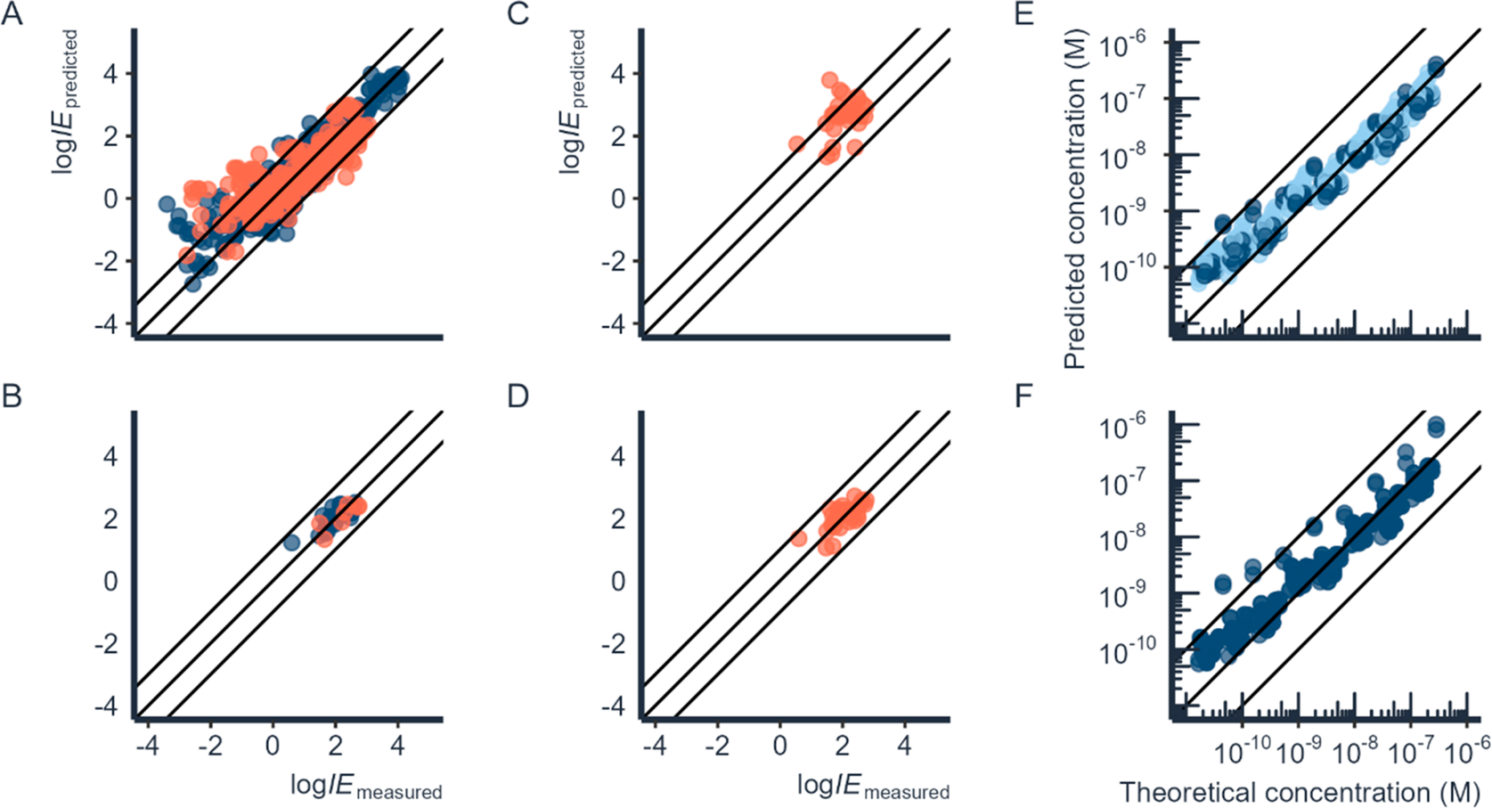
Training (**dark blue**) and test (**orange**) sets of the ESI negative mode prediction model for A) non-PFAS chemicals and B) PFASs. The correlation plots for experimental log *IE* values for PFASs compared to C) predicted log *IE* values using a model without added PFASs and D) predicted log *IE* values obtained from the leave-one-out approach. The correlation between spiked concentrations and E) concentration estimations using the response factor of a homologue chemical that is one −CF_2_– unit smaller (**light blue**) or larger (**dark blue**) compared to the respective suspect and F) concentration estimations using predicted ionization efficiency for the same set of chemicals with a leave-one-out approach.

The new *IE* model was developed on a combined data set including 33 PFASs and 100 compounds measured previously by Liigand et al.^16^ with one chemical - PFOS - occurring in both data sets. The RMSEs over all data points in training and test sets were 0.43 and 0.79 log *IE* units, respectively. This corresponds to fold errors of 2.7 and 6.2 (Figure 3A). The mean, geometric mean, and median prediction errors for the test set were 19.6×, 3.6×, and 2.3×, respectively. For the added PFAS data, the RMSEs of the training and test sets were 0.26 (1.8×) and 0.29 (2.0×) log *IE* units, respectively (Figure 3B). The mean error was 1.9×, the geometric mean error was 1.8×, and the median error was 2.1× on the test set. Out of seven PFASs in the test set, the highest fold prediction error of 2.3× was obtained for 11Cl-PF3OUdS. In comparison, the RMSE obtained by Liigand et al.^16^ for negative mode was 2.0× and 2.3× on training and test sets, respectively. The highest prediction errors for the whole test set were observed for 2-methoxyphenol (831.8×), 4-phenylphenol (563.0×), and 2-nitrophenol (465.8×). These chemicals have low *IE*s (<0 log *IE* units) and are potentially more difficult to model. The training and test set chemicals were joined into one data set, and a new model was trained with previously optimized hyperparameters to further use the model for quantification of suspect PFASs.

To investigate the improvement in model predictions for PFASs, we trained a model based on the original data set used by Liigand et al.^16^ and predicted log *IE*s for each PFAS. The Liigand et al.^16^ data set contained 19 fluorinated compounds but only PFOS from the perfluoroalkyl acids subclass. For comparison, we trained 33 models using the leave-one-out approach to predict log *IE*s for PFASs. The model built on data without adding PFASs resulted in an RMSE of 0.82 log-units (6.6×) on the data set of 33 target PFASs, while using the leave-one-out approach resulted in an RMSE of 0.29 log-units (2.0×). The mean, geometric mean, and median obtained were 11.1×, 4.7×, and 4.8× for the model without PFASs and 2.1×, 1.9×, and 1.6× with the leave-one-out approach, respectively (Figures 3C and 3D). Therefore, a significant improvement was observed in model performance by adding more PFASs to the negative mode *IE*-prediction model (Wilcoxon signed rank test; *p* = 7.54 × 10^–5^). A similar improvement in prediction accuracy has also been reported previously for hydroxylated polychlorinated biphenyls.^43^

Comparing predicted log *IE* to using a response factor of a structurally similar homologue series compound for quantification, the performance of the two approaches was statistically indistinguishable (Wilcoxon signed rank test; *p* = 0.25; Figures 3E and 3F). For additional comparison of quantification results with the machine learning model and homologues with a difference of −C_2_F_4_–, we direct the reader to Figure S2 in the Supporting Information. Nevertheless, predicting *IE* allows us to evaluate the concentration in cases where no homologues are present or if the detected feature cannot be identified at a high level of confidence but multiple candidate structures are present.

### Model Validation

#### Quantification in Spiked Biota Samples

To evaluate the performance of the ionization efficiency predictions in real samples, the models from the leave-one-out approach were used to quantify target PFASs in three liver samples of PW-4, which were spiked with 5 ng of the native PFAS mixture (see Materials and Methods). The predicted concentrations for the targets were compared with the concentrations obtained by quantification using the calibration curve of the respective analytical standards. The mean fold difference between model and target quantification was 2.1×, and in 73% of the cases, quantification with an analytical standard resulted in higher concentrations compared to model quantification (see Figure S17)

#### Prediction for Different Isomers

To assess the effect of isomerization on predicted concentrations, we applied the model to quantify different linear isomers of two CL 3-suspects: Cl-PFNA, using all possible positions of the chlorine substitution along the chain, and ether-PFOS, using all possible positions of the ether linkage. For both substances, we observed that predicted concentrations remain within the same order of magnitude for different linear isomers (Figures S18 and S19); however, branched isomers were not investigated and might show a higher variability in predicted log *IE* (and by extension concentrations) due to inductive effects on the polar headgroup which could lead to changes in p*K*_a_.^44^

### Suspect Quantification with the Final Model

The final model trained on all available *IE* data was used for quantification of CL 2–3 suspects, and the predicted concentrations can be found in Table S10. In the case of CL3 suspects, the structure of the linear isomer has been used for quantification (except for PFECHS). Additionally, in the case of a chlorine substitution, the structure with Cl on the last carbon was used, and for PFESAs, the structure with ether between the first and second carbon was used. For the d-diO/C-diO/K–O PFSA n = 9 suspect, a double bond/diether structure has been selected, and its SMILE is available in Table S10. The applicability of the *IE*-prediction model was confirmed visually with principal component analysis and *t*-distributed stochastic neighbor embedding analysis for all suspects (Figure S20).

The highest average ∑Suspect concentrations were observed in Swedish dolphins (1000 ± 370 ng/g ww/680 ± 250 ng F/g ww), followed by East Greenland pilot whales (17 ± 8 ng/g ww/12 ± 5 ng F/g ww) and West Greenland dolphins (6 ± 3 ng/g ww/4 ± 2 ng F/g ww). One suspect class, n:3 FTCAs, displayed predicted concentrations considerably higher than all the others, with the highest concentrations in Swedish dolphins (average ∑n:3 FTCAs 970 ng/g) and lowest concentrations in pilot whales (average ∑n:3 FTCAs 7.6 ng/g) and absent in Greenlandic dolphins. In this class, the predominant PFAS was 9:3 FTCA (up to 1000 ng/g), followed by 8:3 FTCA (up to 300 ng/g and which had similar concentrations to the target 7:3 FTCA) and 10:3 FTCA (up to 67 ng/g). FTCAs in Swedish dolphins made up ∼95% of the ∑Suspect concentrations. In pilot whale samples, the highest predicted concentrations were observed for 9:3 and 11:3 FTCA (up to 9.2 and 4.8 ng/g, respectively), followed by FHxSA (up to 3.5 ng/g) and perfluoropentadecanoic acid (PFPeDA, up to 2.4 ng/g). In West Greenlandic dolphins, the highest predicted concentrations were found for FPeSA (up to 6.7 ng/g), FHxSA (up to 2 ng/g), and PFPeDA (up to 0.5 ng/g).

### Closing the Fluorine Mass Balance with Machine Learning-Based Quantification

*IE*-based quantification of suspects helped explain significant additional fractions of the EOF (Figure 2, Table S11). The highest predicted concentrations were determined for samples of Swedish dolphins based on the summed fluorine equivalent concentrations of suspects, where an average additional 680 ng F/g could be attributed to suspects. Here, the UEOF after machine learning-based quantification decreased from 53 to 70% to 0–18%. Suspects in pilot whale samples explained an additional 12 ng F/g on average and decreased the UEOF from 13 to 32% to 6–27%. Samples of Greenlandic dolphins already had a closed organofluorine mass balance; nevertheless, some suspects were detected at low concentrations. This is not surprising given the variability of EOF measurements (83% recovery of organofluorine [RSD: 8%]).

The present work offers the possibility of closing the fluorine mass balance in environmental samples in the absence of analytical standards by combining suspect screening with *IE*-based quantification. Nevertheless, a fraction of EOF in some samples remains unexplained. This may arise from poor sensitivity of some PFASs to LC-ESI-MS-based detection, or alternatively, the absence of a tentative structural.

## Supporting Information Available

Additional information on samples, chemicals and reagents, instrumental analysis, PFAS profiles, EICs of homologue series, MS^2^ of suspects, the evaluation of the homologue series approach for quantification, information on molecular descriptors, model evaluation in spiked biota samples and model application domain (PDF)
Table S4 (inclusion list for suspect screening) and Tables S8–S11 (EOF, target and suspect concentrations, fluorine mass balance calculations) (XLSX)
