# Supplementary material for: Closing the Organofluorine Mass Balance in Marine Mammals Using Suspect Screening and Machine Learning-Based Quantification
Source: Environ Sci Technol. 2024 Jan 25;58(5):2458–67. doi: 10.1021/acs.est.3c07220 (PMC10851419; doi:10.1021/acs.est.3c07220)
Supplement: Supplementary file 1 — es3c07220_si_001.pdf [file es3c07220_si_001.pdf]

Supporting Information

Closing the organofluorine mass balance in marine mammals using suspect screening and machine learning-based quantification

Mélanie Z. Lauria,[1*†] Helen Sepman, [1, 2†] Thomas Ledbetter, [1, 2] Merle Plassmann,[1] Anna M. Roos,[3] Malene Simon,[4] Jonathan P. Benskin,[1*#] Anneli Kruve[1, 2#]

[1]Department of Environmental Science, Stockholm University, Svante Arrhenius Väg 8, 10691 Stockholm, Sweden

[2]Department of Materials and Environmental Chemistry, Stockholm University, Svante Arrhenius Väg 16, 106 91, Stockholm, Sweden

[3] Department of Environmental Research and Monitoring, Swedish Museum of Natural History, 104 05 Stockholm, Sweden

[4] Greenland Climate Research Centre, Greenland Institute of Natural Resources, 3900 Nuuk, Greenland

*Corresponding authors:

Melanie.Lauria@aces.su.se

Jon.Benskin@aces.su.se

[†]MZL and HS contributed equally and share first authorship.

[#]AK and JPB contributed equally and share last authorship.

Table of Contents

Table S1: Detailed information on the individual marine mammals sampled.

Table S2: Information for targeted PFAS and isotopically labelled internal standards, recovery and limits of quantification.

Table S3: Eluent information for the ion chromatography part of the EOF analysis by CIC.

Table S4: Inclusion list for suspect screening.

Table S5: The variable importance of 10 most influential PaDEL descriptors in the final model.

Table S6: All target PFAS with homologue series chemicals with -CF_2- difference.

Table S7: All target PFAS with homologue series chemicals with -C_2F_4- difference.

Table S8: Targets concentrations.

Table S9: Extractable Organofluorine (EOF) concentrations.

Table S10: Suspects predicted concentrations.

Table S11: Fluorine mass balance calculations: known and unknown EOF.

Figure S1: Schematic of sample preparation for fluorine mass balance determination.

Figure S2: Comparison between quantification with a homologue series compound of -C_2F_4- difference compared to model approach.

Figure S3: PFAS profiles in marine mammal liver samples.

Figure S4: Extracted Ion Chromatograms of perfluorocarboxylic acids (PFCAs) in liver sample of Swedish dolphin (SD3).

Figure S5: Extracted Ion Chromatograms of perfluorosulfonic acids (PFSAs) in liver sample of Swedish dolphin (SD2).

Figure S6: Extracted Ion Chromatograms of n:3 fluorotelomer carboxylic acids (n:3 FTCAs) in liver sample of Swedish dolphin (SD2).

Figure S7: MS^2 of 7:3 FTCA and 9:3 FTCA in Swedish dolphin liver (SD2).

Figure S8: Extracted Ion Chromatograms of fluorotelomer sulfonic acids (FTSAs) in liver sample of Swedish dolphin (SD2).

Figure S9: MS^2 of 10:2 FTSA in liver of Swedish Dolphin (SD2).

Figure S10: Extracted Ion Chromatograms of fluoroalkane sulphonamides (FASAs) in liver sample of Swedish dolphin (SD1).

Figure S11: MS^2 of FASAs in liver of Swedish Dolphin (SD1 to 3).

Figure S12: Extracted Ion Chromatograms of Cl-PFCAs in liver sample of Greenlandic pilot whale (PW3).

Figure S13 : Cl-PFNA isotopes in pilot whale liver sample (PW1).

Figure S14: Extracted Ion Chromatograms of ether-PFSAs in liver sample of Swedish dolphin (SD2).

Figure S15: Extracted Ion Chromatograms of unknown homologue $C_nF_{2n-9}H_9NO_4SH$ class in liver sample of Swedish dolphin (SD2).

Figure S16: MS^2 of [C13F17H9NO4S]- in liver samples of SD2 and SD3.

Figure S17: Target and model quantification in three spiked liver samples.

Figure S18: Predicted concentration in pg/ul for different isomers of Cl-PFNA.

Figure S19: Predicted concentration in pg/ul for different isomers of ether-PFOS.

Figure S20: Model application domain.

Chemical and reagents

Acetonitrile (≥99%, Chromasolv™) was from Honeywell (France). Envicarb (Supelclean™) came from Sigma Aldrich. Stainless steel beads came from Next Advance©. Argon and oxygen gases used during CIC analysis were of purity grade 5.0 milliq, and fluoride standard (1000 mg/L) was from Thermo Scientific. Water was purified with a Millipore purification system that had a resistance of <18 MΩ/cm (Milli-Q water). Ammonium acetate and methanol (99.8%, LiChrosolv®) were from Merck (Darmstadt, Germany).

Sample preparation for organofluorine mass balance

Approximately 0.5 g of liver was fortified with 4 ml of acetonitrile together with 7-8 beads (stainless steel ø 4.8 mm) and homogenized in a bead blender (SPEX SamplePrep 1600 MiniG®) for 5 min at 1500 rpm. Samples were then centrifuged at 2000 rpm for 5 min (Centrifuge 5810, Eppendorf, Hamburg), and the supernatant was transferred to a new 13 mL PP tube. The extraction was repeated by adding another 4 mL of acetonitrile, vortexing and centrifuging again. The new supernatant was added to the existing tube containing the previous supernatant. Combined extracts were concentrated to 1 mL under a stream of nitrogen in a water bath at 40 °C (TurboVap LV Evaporator, Biotage). Concentrated extracts were then weighed and added to a 1.7 ml Eppendorf tube containing 25 mg EnviCarb and 50 µl acetic acid. The samples were vortexed and centrifuged for 10 min at 10 000 rpm (Galaxy 14D, Microcentrifuge, VWR), then split into two aliquots of 250 uL each:

Aliquot 1: 250 uL of supernatant destined for UPLC-HRMS analysis was transferred to another Eppendorf tube and spiked with 1 ng of IS mix. 250 µl of NH_4OAc (4 mM in water) was added to the extracts as a buffer, vortexed and stored at -20 °C. Upon analysis, extracts were adjusted to room temperature, vortexed, and transferred to LC vials.

Aliquot 2: 250 uL of supernatant destined for CIC analysis was transferred to another Eppendorf tube and stored at -20 °C. ISs are not added to this aliquot since the fluorine in the isotopically labelled standards would influence the results. Upon analysis, extracts were adjusted to room temperature, and vortexed if needed.

Extractable organofluorine analysis

EOF measurements were carried out with a Thermo-Mitsubishi CIC using a previously described method. Extracts (200 uL per sample) were transferred into ceramic containers ("boats″) which contained glass wool for fluid dispersion. Boats were prebaked prior to analysis after being cleaning with soap, water and a basic solution. Samples were combusted slowly in a horizontal furnace (HF-210, Mitsubishi) at 1100°C under a flow of oxygen (400 mL/min), argon (200 mL/min), and water vapor mixed with argon (100 mL/min) for 5 minutes. Combustion gases were absorbed by MilliQ water during the combustion process with a gas absorber unit (GA-210, Mitsubishi). 200 uL of the absorption solution was then injected onto an ion chromatograph (Dionex Integration HPIC, Thermo Fisher Scientific) equipped with an anion exchange column (2 x 50 mm guard column (Dionex IonPac AS19-4um) and 2 x 250 mm analytical column (Dionex IonPac AS19-4um) operated at 35°C. A gradient of aqueous hydroxide mobile phase was ramped from 8mM to 100 mM at 0.25 mL/min (Table S3) and fluoride was detected using a conductivity detector.

A standard calibration curve was prepared with a range of 0.05 to 100 ug F/mL and subsequently used for quantification within the linear range (R^2>0.97). Mean fluoride concentration from procedural blanks was subtracted from samples before quantification. Boats were prebaked prior to analysis after being cleaning with soap, water and a basic solution.

Method detection limit (MDL) was calculated using the standard deviation of F concentrations from procedural blanks (n=3, each batch).

Target and suspect screening analysis

Sample extracts (5 µL) were injected onto a Dionex Ultimate 3000 RS UHPLC system (Thermo Scientific) equipped with an ethylene bridge hybrid (BEH) C_{18} column was used (1.7 um, 50 x 2.1 mm, Waters). Mobile phase A consisted of 90% H_2O with 10% acetonitrile, mobile phase B consisted of 99% acetonitrile and 1% H_2O, both with ammonium acetate at 2 mM (0.1542 g/L). The following gradient elution program was used at a flowrate of 0.4 mL/min: 0-0.5 minutes, 10% B; 0.5-8 minutes, 80% B; 8-8.1 minutes, 100% B; 8.1-11 minutes, 100% B; 11-11.1 minutes, 10% B; 11.1-13 minutes, 10% B. The autosampler sample tray was cooled to 15°C and the column temperature was 30°C.

A Q-Exactive™ ultra high mass resolution (UHMR) hybrid Quadrupole-Orbitrap™ mass spectrometer was used with alternating Full Scan (FS) Data-Dependent (DD) MS^2 mode. In FS the scan range was 200-1800 *m/z* in resolution was 120 000 using the full width at half maximum definition for an *m/z* of 200. The Automatic Gain Control (AGC) target was 3 x 10^6. Maximum inject time was 250 ms. Electrospray ionization settings were set to negative mode, sheath gas flowrate was 30 arbitrary units (AU), aux gas flowrate was 10 AU, sweep gas flowrate was 0 AU, spray voltage was 3.7 kV, capillary temp was 350 °C, S-lens RF level was 50 AU, and aux gas heater temp was 350 °C.

For each analyte, the concentration of the lowest calibration standard with at least a 10000 counts per second (cps) and signal to noise ratio higher than 3 was used as the method limit of quantification (method LOQ). The mean extract volume ($\overline{extract}$ = 1045.8 uL) and mean sample mass ($\overline{sample\ mass}$ = 0.54 g) were used to determine the LOQ in liver samples.

$$LOQ\ \left(\frac{ng}{g}\right) = cal_{lowest}(\frac{ng}{\mu l}) \cdot \frac{\overline{extract(\mu l)}}{\overline{sample\ mass\ (g)}} \qquad (eq. 1)$$

If a compound was found to be present in the blanks, its concentration in blanks plus three times the standard deviation in the blanks was used in place of the concentration in the lowest suitable calibration level to determine the LOQ with equation (1).

Confidence levels

Confidence Levels (CLs) were assigned to suspects according to the scale proposed by Schymanski et al (see overview in the SI).[23] Briefly, CL 1 is a confirmed structure by comparison with a reference analytical standard, CL 2 is a probable structure and it is subdivided in CL 2a, which is assigned when the MS^2 is matched with literature or a library, and CL 2b, which in this study has been assigned to those suspects that are part of homologue series for which retention times increase with increasing chain length, but none or only a few fragments are observed. CL 3 is a tentative candidate for which one or more possible structures are proposed. CL 4 refers to a unequivocal molecular formula and CL 5 to and exact m/z of interest.

Quality control

A suite of QC procedures was included to ensure the accuracy and precision of EOF and target PFAS data, including spiked liver (with native PFAS and inorganic fluorine, both n=3), procedural blanks (n=3), and analysis of certified reference materials (CRM, n=3). In all cases, QC samples were analysed in the same manner as real samples and background subtraction (using unspiked liver for spiked liver samples experiments and procedural blanks for liver samples) was performed prior to calculations. Direct combustion (i.e. without extraction) of CRM BCR-461 (fluorine in clay) produced concentrations that were highly consistent with the reference value (579 ± 84 µg F/g, certified 568 ± 60 µg F/g), demonstrating accuracy and precision of the CIC method. The inorganic fluorine spike/recovery experiment, which involved subsamples (0.5 g) of liver prepared with and without a 250 ng NaF spike demonstrated effective removal of inorganic fluorine during extraction (average recovery 0%, RSD 11%), consistent with prior results using this method.[10,24] Finally, non-IS corrected recoveries of organofluorine (determined by CIC) and target PFAS (determined by LC-HRMS) using subsamples (0.5 g) of liver prepared with and without a 5 ng native PFAS mixture (equivalent to 155.7 ng of F) were largely acceptable (average recoveries of 83% [RSD 8%] for EOF by CIC and 80-150% for most targets by LC-HRMS). The exception was for a handful of targets measured by LC-HRMS (i.e. PFUnDA, PFHxDA, FTCAs, 4:2 and 8:2 FTSAs, MeFOSA, EtFOSA and FOSAA) which displayed higher or lower recoveries, possibly due to a non-exactly matched internal standard or matrix suppression and enhancement processes. Detailed values can be found in Table S2, together with limits of quantification for targets.

Cleaning of descriptor for modelling

First, all 1449 (1444 PaDEL and five eluent) descriptors were confirmed not to include more than 10 missing values per descriptor. Next, all descriptors with variance above a cut-off value of 80/20 (the ratio of first over second most common value) were removed, resulting in 796 descriptors. Lastly, the correlation between descriptors was calculated and for descriptors with a pair-wise correlation higher than 0.75, the descriptor with a higher mean absolute correlation was removed. The resulting 124 descriptors were used for further modelling.

Table S1: Detailed information on the marine mammals sampled. Empty cells indicate unknown information. NMR: Swedish museum of natural history; ACES: department of environmental science at Stockholm University, CITES: Convention on International Trade in Endangered Species of Wild Fauna and Flora.

	**Name in paper**	**Greenland Nr**	**Date**	**Sex**	**Length (m)**	**Age**	**Organ**	**ACES nr**	**CITES Nr**
**White beaked dolphins, Maniitsoq, West Greenland**	GD1	La_88	Fall 2018	male			liver	PAX20/0164	20GL1717529
	GD2	La_89	Fall 2018	male			liver	PAX20/0166	
	GD3	La_90	Fall 2018	male			liver	PAX20/0168	
	GD4	La_92	Fall 2018	male			liver	PAX20/0170	
	GD5	La_93	Fall 2018	male			liver	PAX20/0172	
**Long-finned pilot whales, Tasiilaq, East Greenland**	PW1	Gm_01	2018-08-20	female	4-5	18	liver	PAX20/0174	20GL1717532
	PW2	Gm_02	2018-08-20	female	4-5	9	liver	PAX20/0176	
	PW3	Gm_10	2018-09-11	female	3.8		liver	PAX20/0180	
	PW4	Gm_11	2018-09-11	female	4	13	liver	PAX20/0182	
	PW5	Gm_15	2018-09-11	female	3-4		liver	PAX20/0184	
	**Name in paper**	**NMR Nr**	**Date**	**Sex**	**Length (m)**	**Age**	**Organ**	**ACES Nr**	**Locality**
**White beaked dolphins, Sweden**	SD1	A2009/05048	2007-10-12	female	2.7	Adult, old	liver	PAX20/0225	Ängelholm, Skåne
	SD2	A2009/05104	2009-02-01		1.5	Juvenile	liver	PAX20/0227	Kungsbacka, Halland
	SD3	A2015/05634	2008-01-28	female	2		liver	PAX20/0229	Ängelholm, Skåne

Table S2: Information for targeted PFAS. Some compounds did not have a direct internal standard therefore internal standard with the closest possible structure was used. IS: internal standard, SD: standard deviation, LOQ: limit of quantification.

* : indicates targets not found in any liver sample.

**Class**	**Analyte**	**IS**	**Recovery ± SD (%)**	**LOQ (ng/g)**
PFCAs	PFPeA*	$^{13}C_5$-PFPeA	118 ± 1	0.155
	PFHxA	$^{13}C_2$-PFHxA	97 ± 3	0.045
	PFHpA	$^{13}C_4$-PFHpA	95 ± 1	0.045
	PFOA	$^{13}C_4$-PFOA	102 ± 3	0.047
	PFNA	$^{13}C_5$-PFNA	93 ± 12	0.047
	PFDA	$^{13}C_2$-PFDA	93 ± 15	0.047
	PFUnDA	$^{13}C_2$-PFUnDA	48 ± 44	0.047
	PFDoDA	$^{13}C_2$-PFDoDA	98 ± 12	0.047
	PFTrDA	$^{13}C_2$-PFDoDA	81 ± 56	0.047
	PFTeDA	$^{13}C_2$-PFDoDA	146 ± 14	0.047
	PFHxDA*	$^{13}C_2$-PFDoDA	193 ± 4	0.045
PFSAs	PFBS*	$^{18}O_2$-PFHxS	106 ± 5	0.141
	PFHxS	$^{18}O_2$-PFHxS	145 ± 6	0.149
	PFOS	$^{13}C_4$-PFOS	81 ± 24	0.043
	PFDS	$^{13}C_4$-PFOS	99 ± 5	0.153
n:3 FTCAs	3:3 FTCA*	$^{13}C_4$-PFOA	42 ± 1	0.556
	5:3 FTCA	$^{13}C_4$-PFOA	56 ± 4	0.047
	7:3 FTCA	$^{13}C_4$-PFOA	66 ± 3	0.161
n:2 FTSAs	4:2 FTSA*	$^{13}C_2$-6:2 FTSA	42 ± 1	0.048
	6:2 FTSA*	$^{13}C_2$-6:2 FTSA	111 ± 1	0.048
	8:2 FTSA	$^{13}C_2$-6:2 FTSA	57 ± 2	0.048
FASAs	FOSA	$^{13}C_8$-FOSA	93 ± 15	0.047
	MeFOSA*	D_3-MeFOSA	155 ± 9	0.047
	EtFOSA*	D_5-EtFOSA	175 ± 4	0.047
FASAAs	FOSAA*	D_3-MeFOSAA	157 ± 2	0.047
	MeFOSAA*	D_3-MeFOSAA	100 ± 1	0.048
	EtFOSAA*	D_5-EtFOSAA	90 ± 3	0.047
diPAPs	6:2 diPAP*	$^{13}C_4$-6:2 diPAP	96 ± 2	0.047
	6:2/8:2 diPAP*	$^{13}C_4$-6:2 diPAP	83 ± 0.3	0.047
	8:2 diPAP*	$^{13}C_4$-8:2 diPAP	98 ± 4	0.162
Cl-PFESAs	9Cl-PF3ONS*	$^{13}C_4$-PFOS	93 ± 2	0.047
	11Cl-PF3OUdS*	$^{13}C_4$-PFOS	94 ± 4	0.047
PFECAs	ADONA*	$^{13}C_4$-PFOS	96 ± 3	0.047
Recovery Standard		$^{13}C_8$-PFOA		
		$^{13}C_8$-PFOS		

Table S3: Eluent information for the ion chromatography part of the EOF analysis by CIC.

Time (min)	Concentration OH^- (mM)
0	8
9	8
11	100
14	100
14,1	8
20	8

Table S4: Inclusion list for suspect screening.

Refer to accompanying Excel file.

Table S5: The relative variable importance and details of the 10 most influential PaDEL descriptors for the final model based on combined training and test set data.

Feature	Relative importance	Descriptor class	Description
AATS6e	100.0	Auto correlation descriptor	Average Broto-Moreau autocorrelation - lag 6 / weighted by Sanderson electronegativities
ASP-6	66.5	ChiPath descriptor	Average simple path, order 6
VP-7	38.5	ChiPath Descriptor	Valence path, order 7
VR2_Dzi	36.7	Barysz Matrix descriptor	Normalized Randic-like eigenvector-based index from Barysz matrix / weighted by first ionization potential
BCUTw-1l	35.0	BCUT Descriptor	nhigh lowest atom weighted BCUTS
ALogP	22.6	ALOGP descriptor	Ghose-Crippen LogKow
pH.aq.	22.0	Eluent descriptor	Aqueous pH
VR2_Dzs	9.7	Barysz Matrix descriptor	Normalized Randic-like eigenvector-based index from Barysz matrix / weighted by I-state
polarity_index	9.7	Eluent descriptor	Polarity index
GATS5c	9.3	Auto correlation descriptor	Geary autocorrelation - lag 5 / weighted by charges

Table S6: All target PFAS with homologue series chemicals with -CF_2- difference.

Compound subject to quantification	Homologue series compound used in quantification	Smaller or larger by -CF_2-
PFDA	PFNA	smaller
	PFUnDA	bigger
PFDoDA	PFTrDA	bigger
	PFUnDA	smaller
PFHpA	PFHxA	smaller
	PFOA	bigger
PFHxA	PFHpA	bigger
	PFPeA	smaller
PFNA	PFDA	bigger
	PFOA	smaller
PFOA	PFHpA	smaller
	PFNA	bigger
PFPeA	PFHxA	bigger
PFTeDA	PFTrDA	smaller
PFTrDA	PFDoDA	smaller
	PFTeDA	bigger
PFUnDA	PFDA	smaller
	PFDoDA	bigger

Table S7: All target PFAS with homologue series chemicals with $-C_2F_4-$ difference.

Compound subject to quantification	Homologue series compound used in quantification	Smaller or larger by $-C_2F_4-$
11Cl-PF3OUdS	9Cl-PF3ONS	smaller
4:2 FTS	6:2 FTS	bigger
6:2 diPAP	6:2/8:2 diPAP	bigger
6:2 FTS	4:2 FTS	smaller
	8:2 FTS	bigger
6:2/8:2 diPAP	6:2 diPAP	smaller
	8:2 diPAP	bigger
8:2 diPAP	6:2/8:2 diPAP	smaller
8:2 FTS	6:2 FTS	smaller
9Cl-PF3ONS	11Cl-PF3OUdS	bigger
7:3 FTA	5:3 FTA	smaller
5:3 FTA	7:3 FTA	bigger
	3:3 FTA	smaller
3:3 FTA	5:3 FTA	bigger
PFBS	PFHxS	bigger
PFDA	PFDoDA	bigger
	PFOA	smaller
PFDoDA	PFDA	smaller
	PFTeDA	bigger
PFDS	PFOS	smaller
PFHpA	PFNA	bigger
	PFPeA	smaller
PFHxA	PFOA	bigger
PFHxDA	PFTeDA	smaller
PFHxS	PFBS	smaller
	PFOS	bigger
PFNA	PFHpA	smaller
	PFUnDA	bigger
PFOA	PFDA	bigger
	PFHxA	smaller

PFOS	PFDS	bigger
	PFHxS	smaller
PFPeA	PFHpA	bigger
PFTeDA	PFDoDA	smaller
	PFHxDA	bigger
PFTriDA	PFUnDA	smaller
PFUnDA	PFNA	smaller
	PFTriDA	bigger

Table S8: Target concentrations in ng/g, sum of targets concentrations (∑PFAS (ng/g)), targets concentrations in fluorine equivalents and sum of targets concentrations in fluorine equivalents (∑FPFAS (ng F/g)).

Refer to accompanying Excel file.

Table S9: Extractable organofluorine concentrations in ng F/g.

Refer to accompanying Excel file.

Table 10: Suspects concentrations in ng/g, sum of suspects concentrations (∑Suspects (ng/g)), Suspects concentrations in ng F/g and sum of suspects concentrations in fluorine equivalents ($\sum_F$Suspects (ng F/g)).

Refer to accompanying Excel work book.

Table S11: Fluorine mass balance calculations: known and unknown EOF.

Refer to accompanying Excel work book.

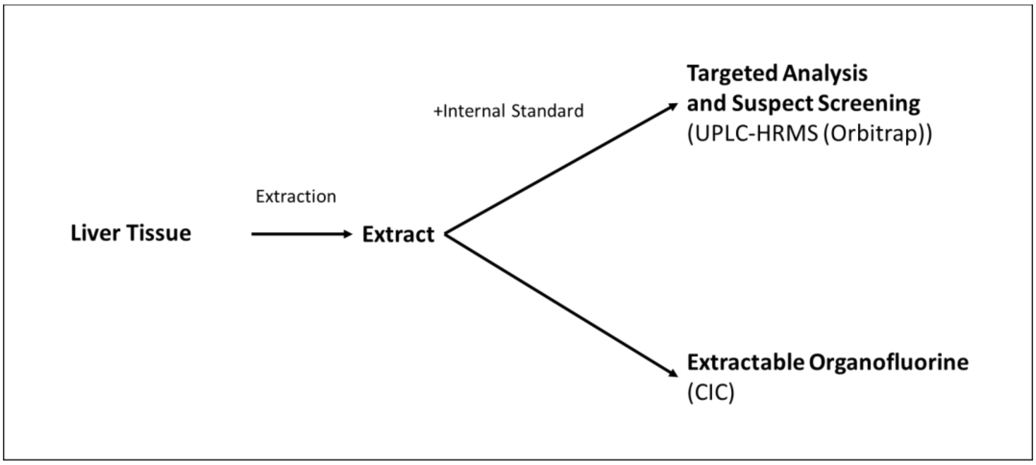

Figure S1: Schematic of sample preparation for fluorine mass balance determination.

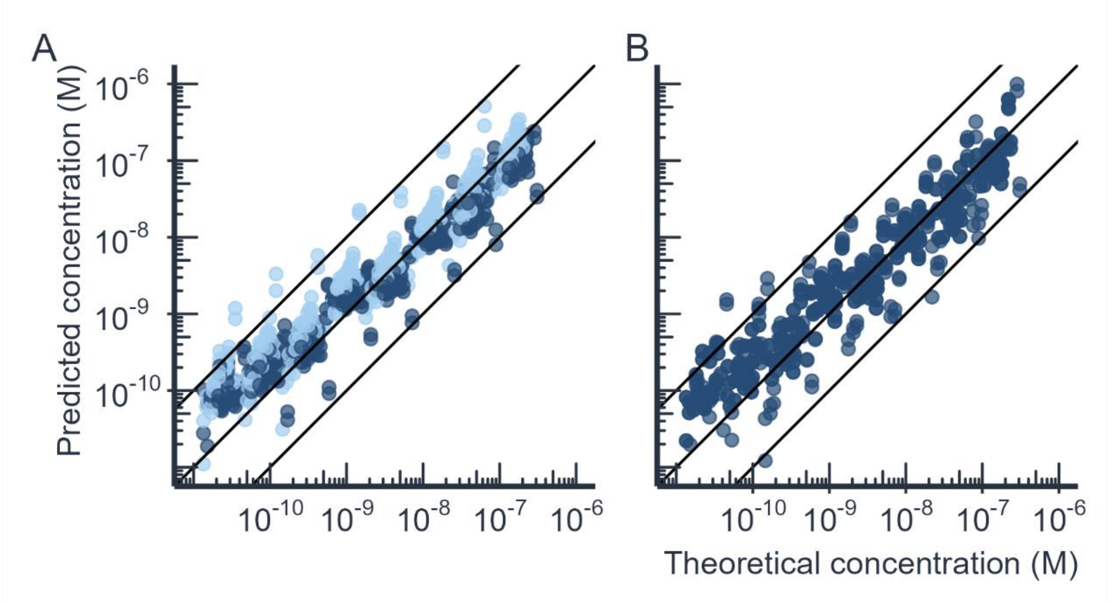

Figure S2: Comparison between quantification with A) a homologue of -C_2F_4- unit smaller (**light blue**) or larger (**dark blue**) compared to respective suspect and B) concentration estimations using predicted ionization efficiency for the same set of chemicals with leave-one-out approach. The mean fold errors in concentrations were 2.6 and 3.1 when smaller or larger homologue was used for quantification, respectively. The mean fold error for quantification with model was 2.7 compared to mean fold error over all homologues of 2.8. The difference between quantification with model compared to using a homologue series approach was statistically insignificant (Wilcoxon signed rank test; p = 0.26). Similarly, the 10 target PFAS in a homologue series with difference in one -CF_2- unit resulted in a mean fold concentration error of 2.2×, while the log*IE* modelling approach had a mean fold concentration error of 2.5×. The quantification with a smaller homologue (fold error of 2.2×) and larger homologue (fold error of 2.3×) performed essentially the same (Figure 4E and 4F).

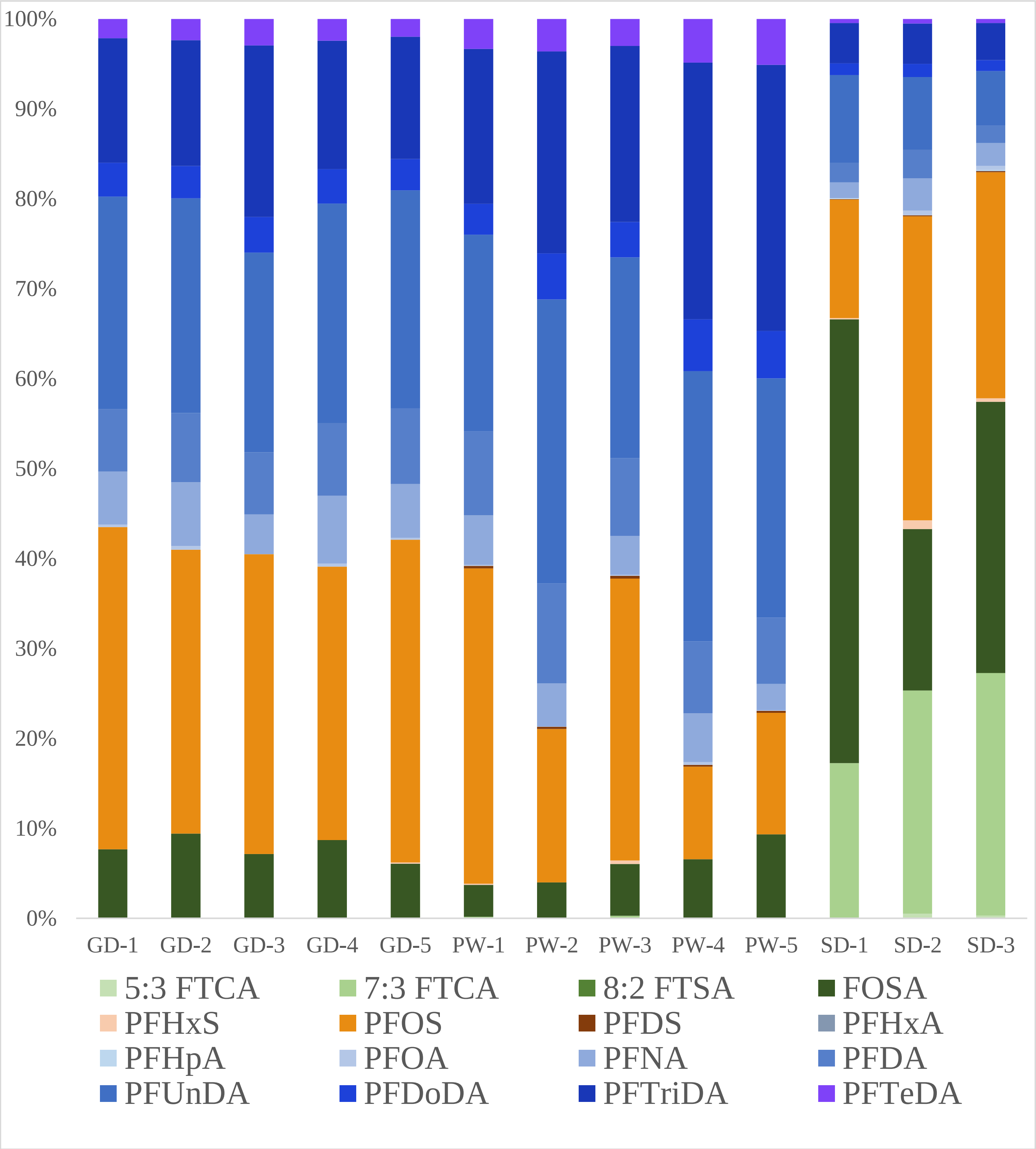

Figure S3: PFAS profiles in marine mammal liver samples. GD: Greenlandic dolphins, PW: Greenlandic pilot whales, SD: Swedish dolphins. Profiles are similar among all samples, with the notable difference of 7:3 FTCA being present only in SD as well as FOSA having a higher percentage in these samples.

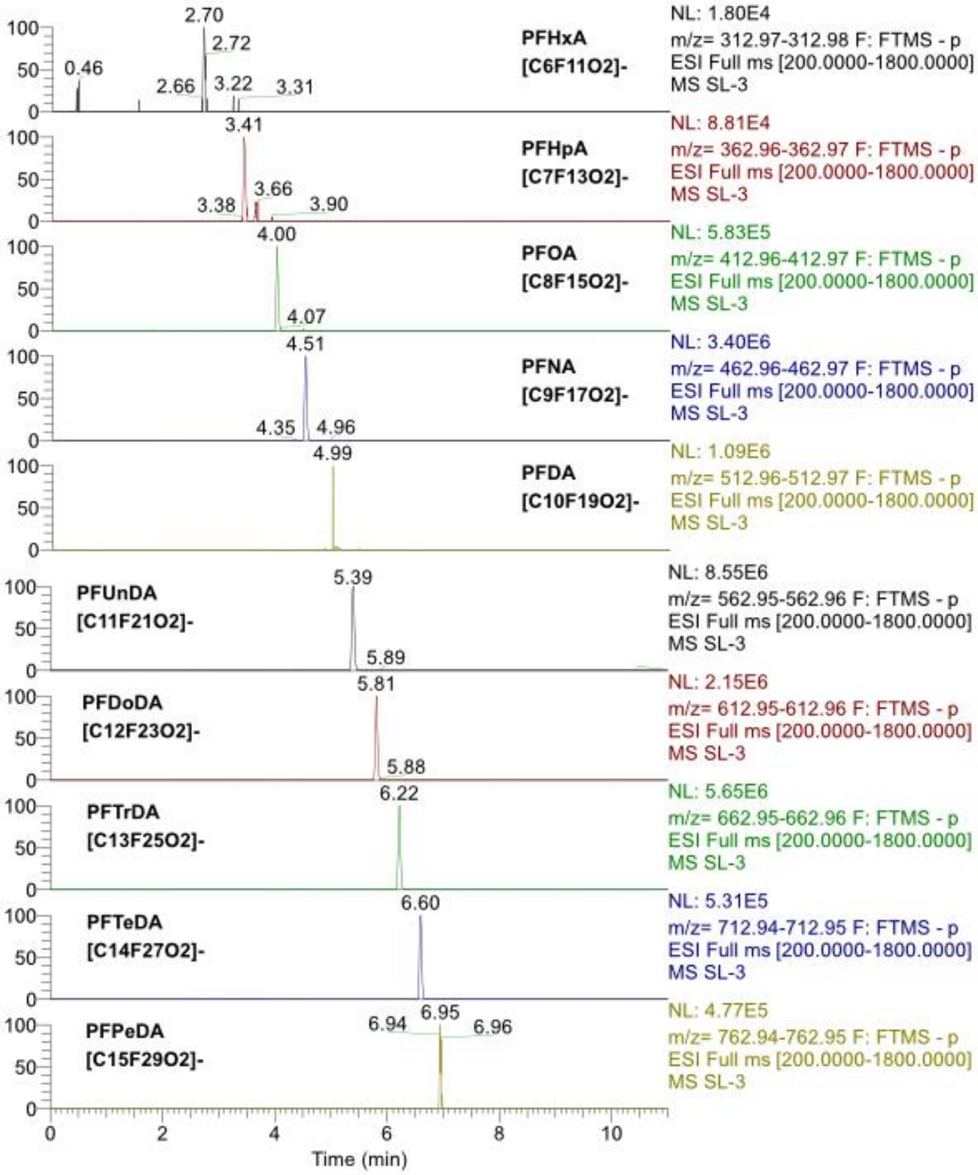

Figure S4: Extracted Ion Chromatograms of perfluorocarboxylic acids (PFCAs) in liver sample of Swedish dolphin (SD3).

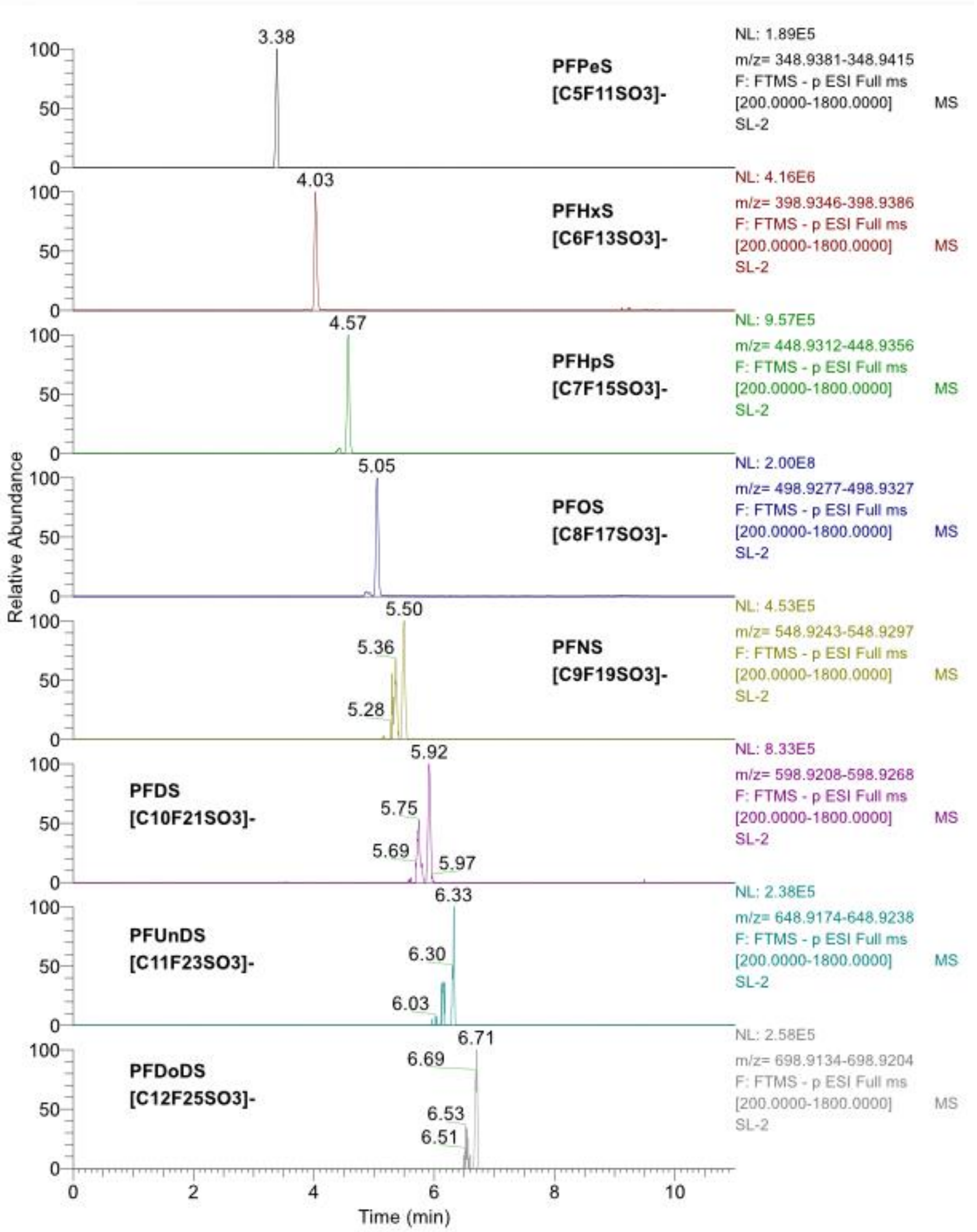

Figure S5: Extracted Ion Chromatograms of perfluorosulfonic acids (PFSAs) in liver sample of Swedish dolphin (SD2).

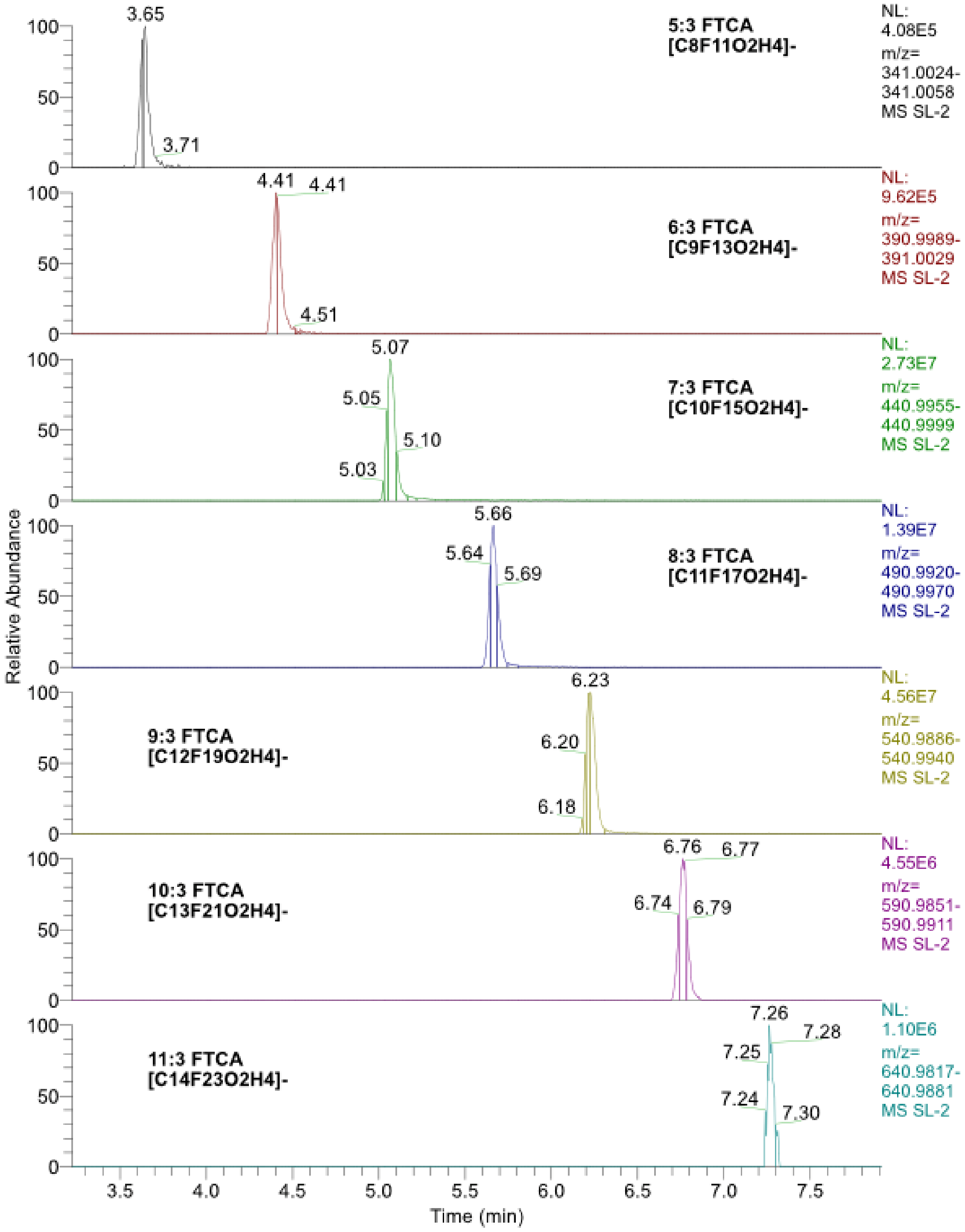

Figure S6: Extracted Ion Chromatograms of n:3 fluorotelomer carboxylic acids (n:3 FTCAs) in liver sample of Swedish dolphin (SD2). Single scan dropouts are due to the triggering of MS/MS scans.

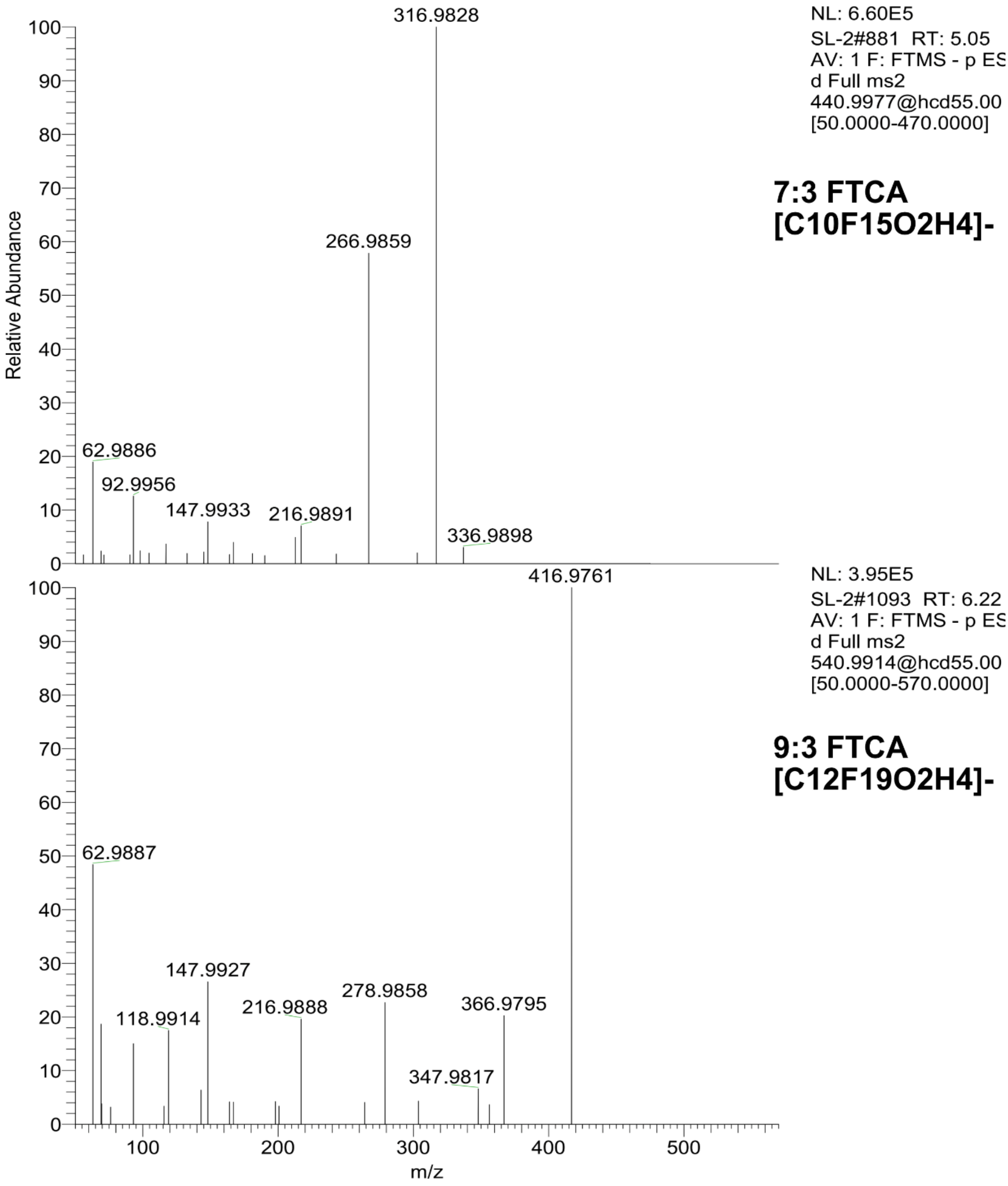

Figure S7: MS^2 of 7:3 FTCA and 9:3 FTCA in Swedish dolphin liver (SD2).

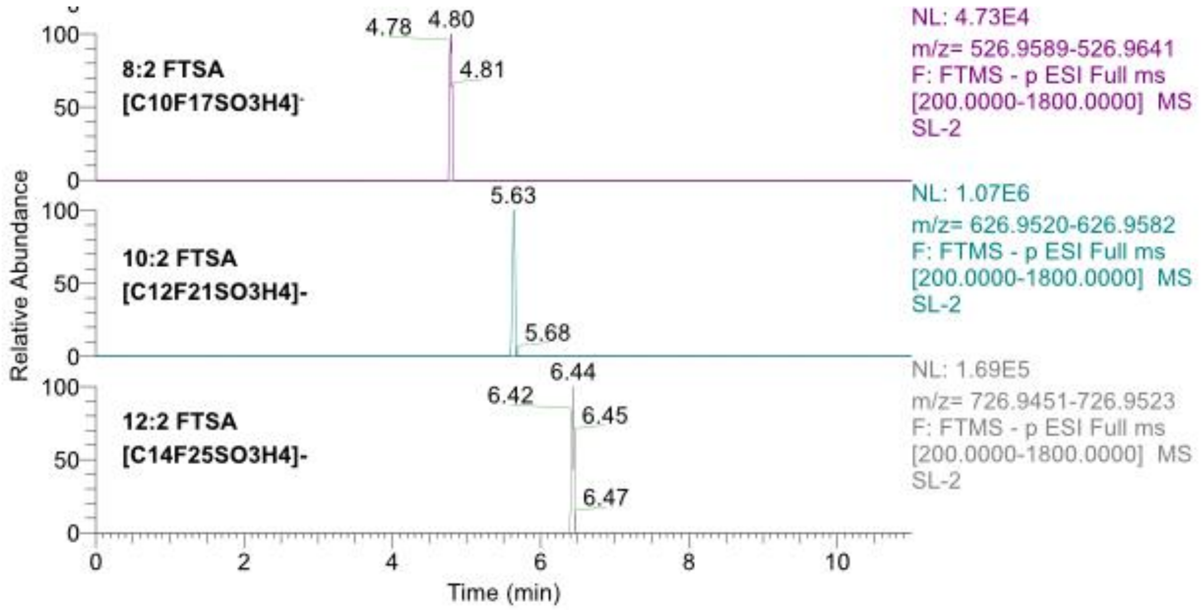

Figure S8: Extracted Ion Chromatograms of fluorotelomer sulfonic acids (FTSAs) in liver sample of Swedish dolphin (SD2).

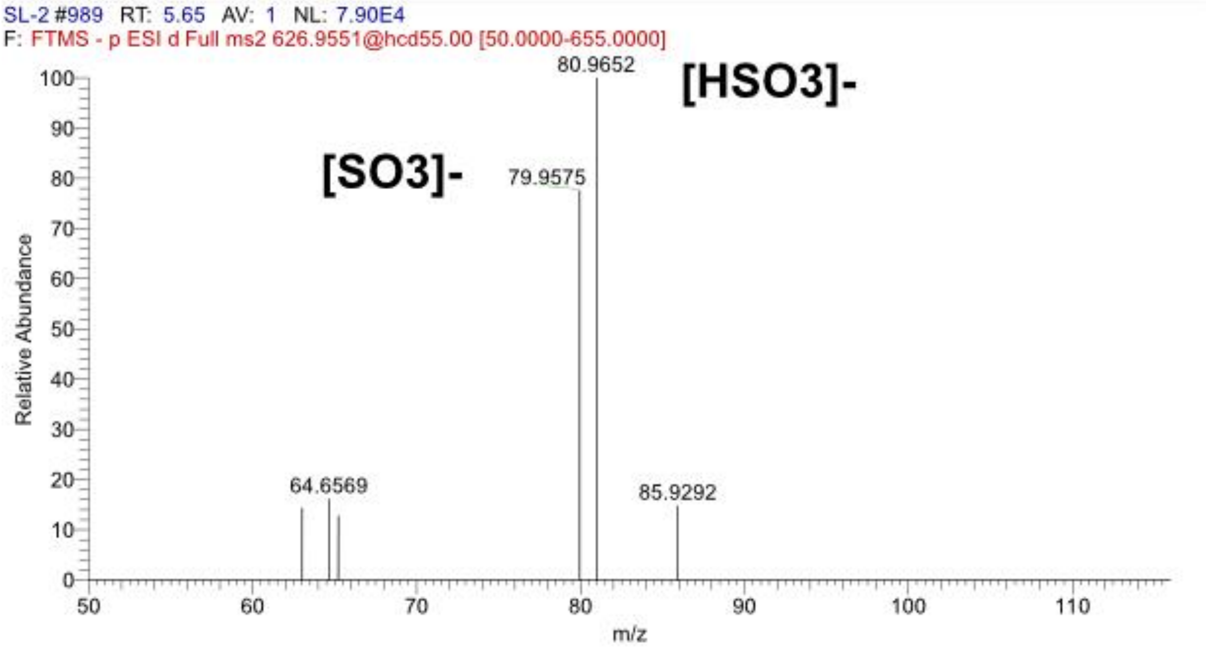

Figure S9: MS^2 of 10:2 FTSA in liver of Swedish Dolphin (SD2).

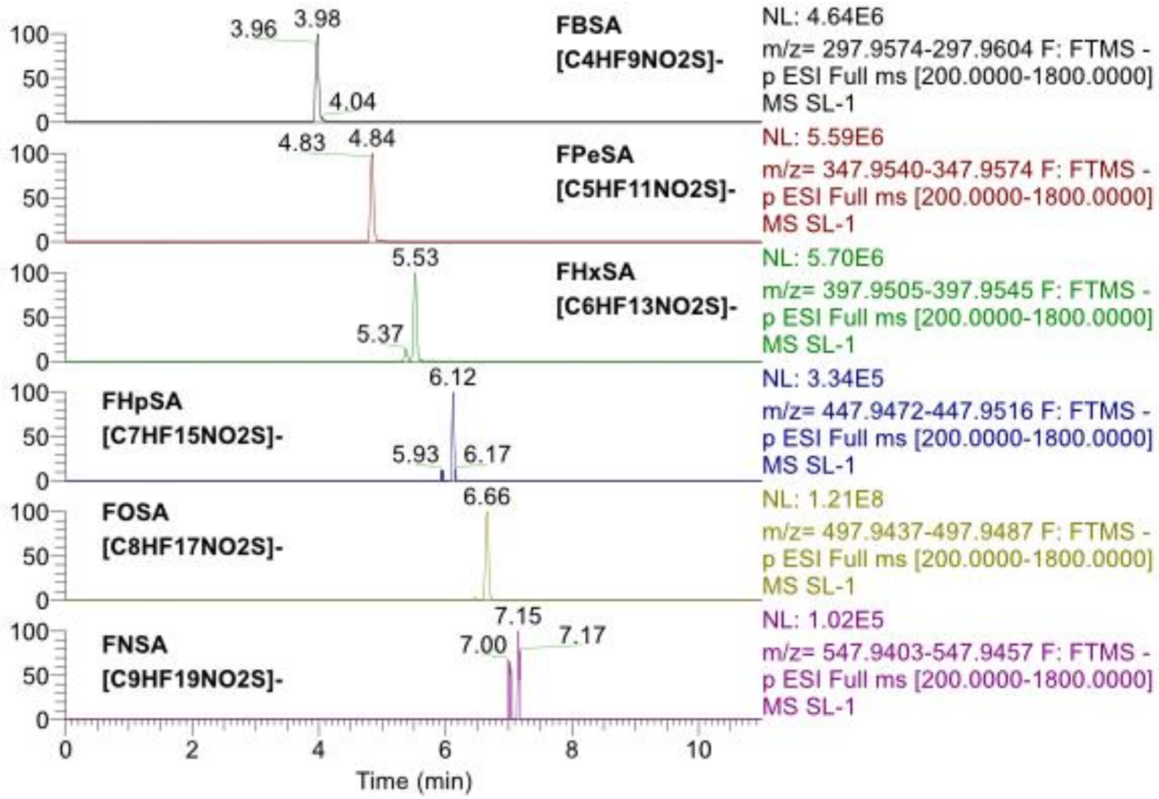

Figure S10: Extracted Ion Chromatograms of fluoroalkane sulphonamides (FASAs) in liver sample of Swedish dolphin (SD1).

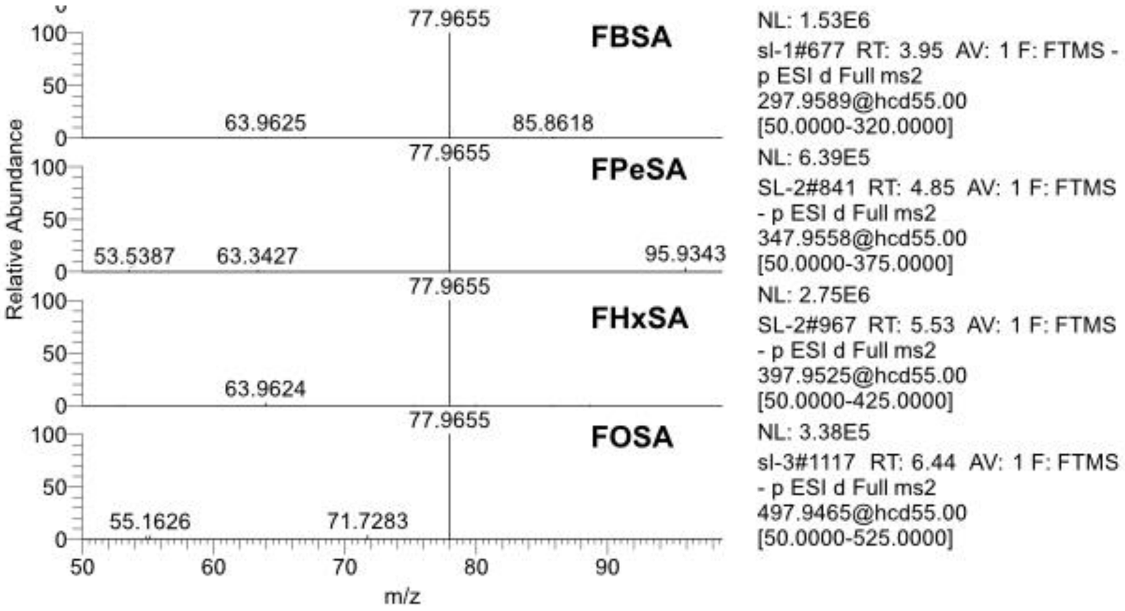

Figure S11: MS^2 of FASAs in liver of Swedish Dolphin (SD1 to 3). With characteristic $[NSO_2]^-$ fragment.

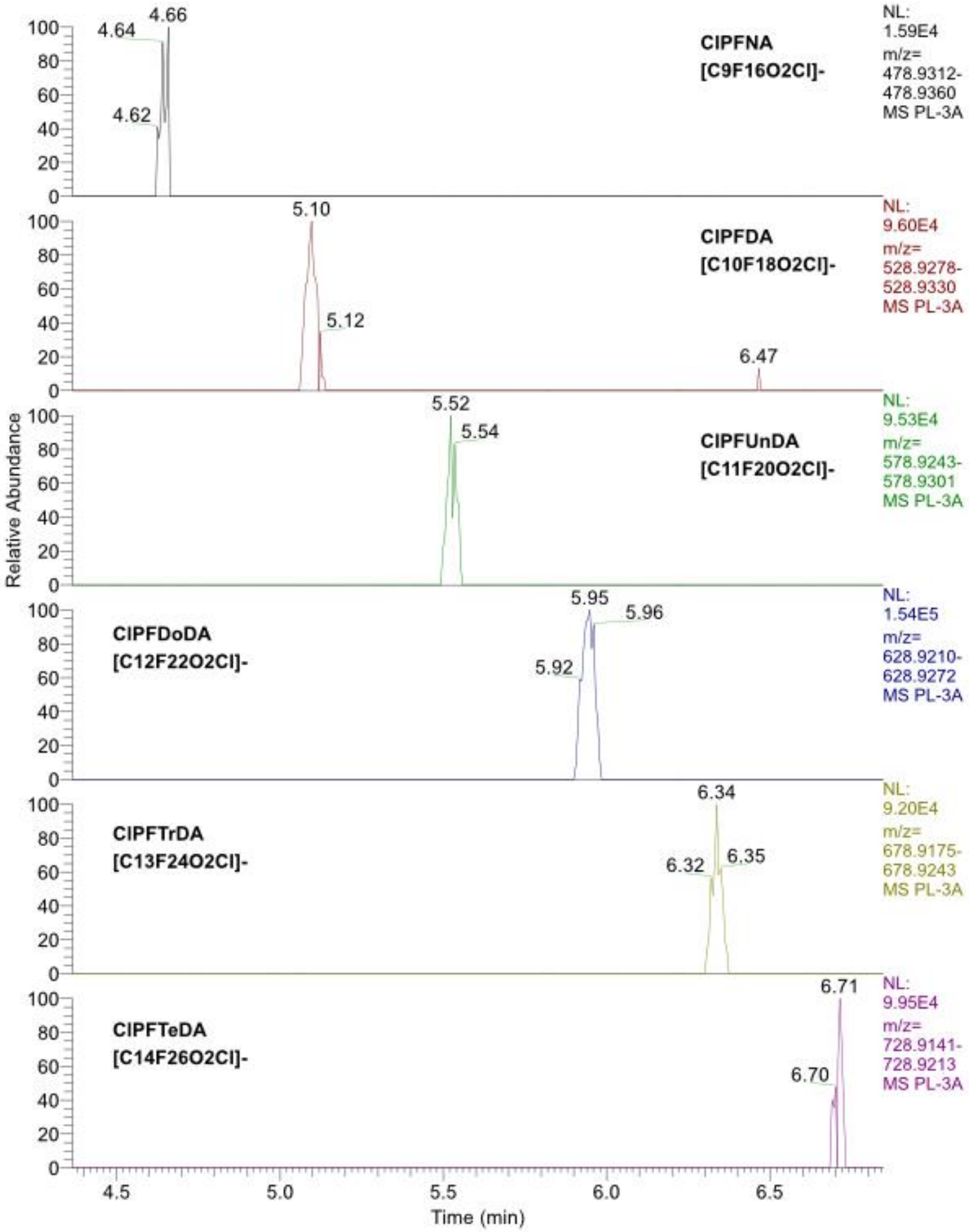

Figure S12: Extracted Ion Chromatograms of Cl-PFCAs in liver sample of Greenlandic pilot whale (PW3).

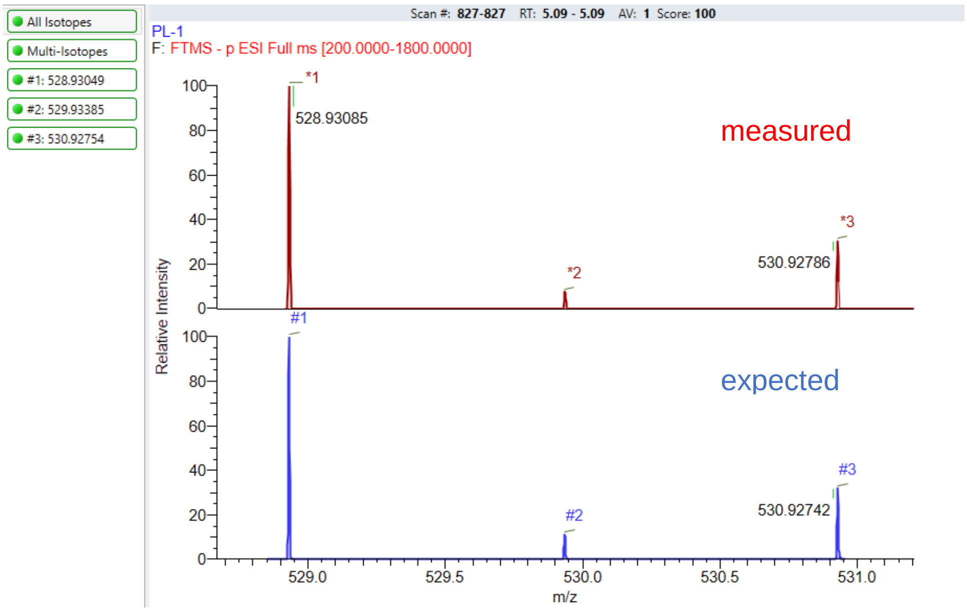

Figure S13 – Cl-PFNA isotopologues in pilot whale liver sample (PW1). Number 3 represents the ^{37}Cl-isotopologue.

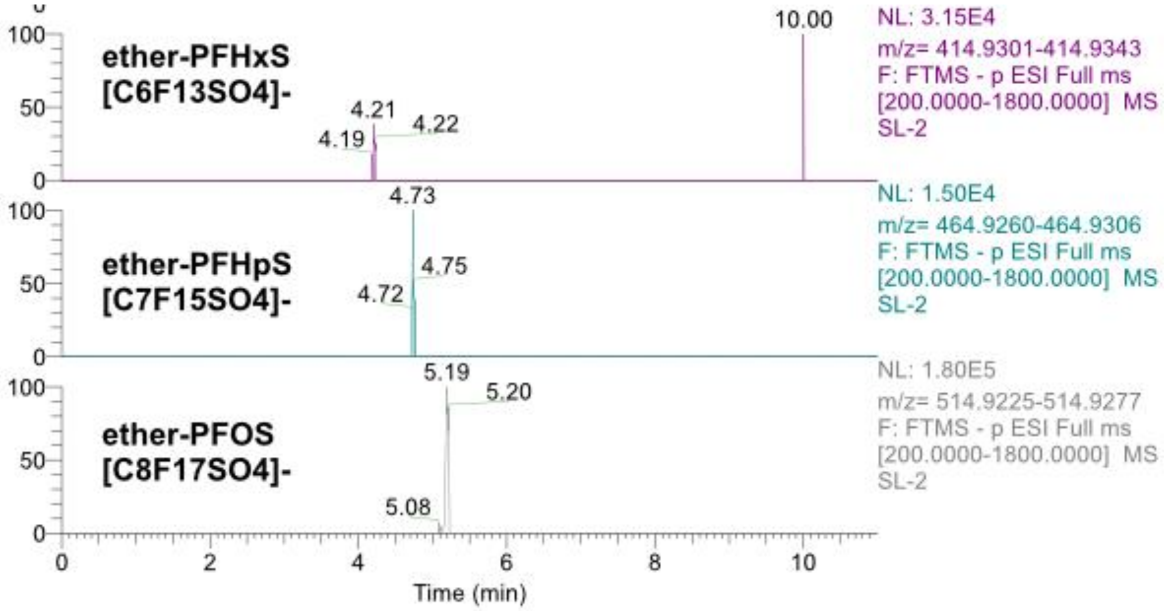

Figure S14: Extracted Ion Chromatograms of ether-PFSAs in liver sample of Swedish dolphin (SD2).

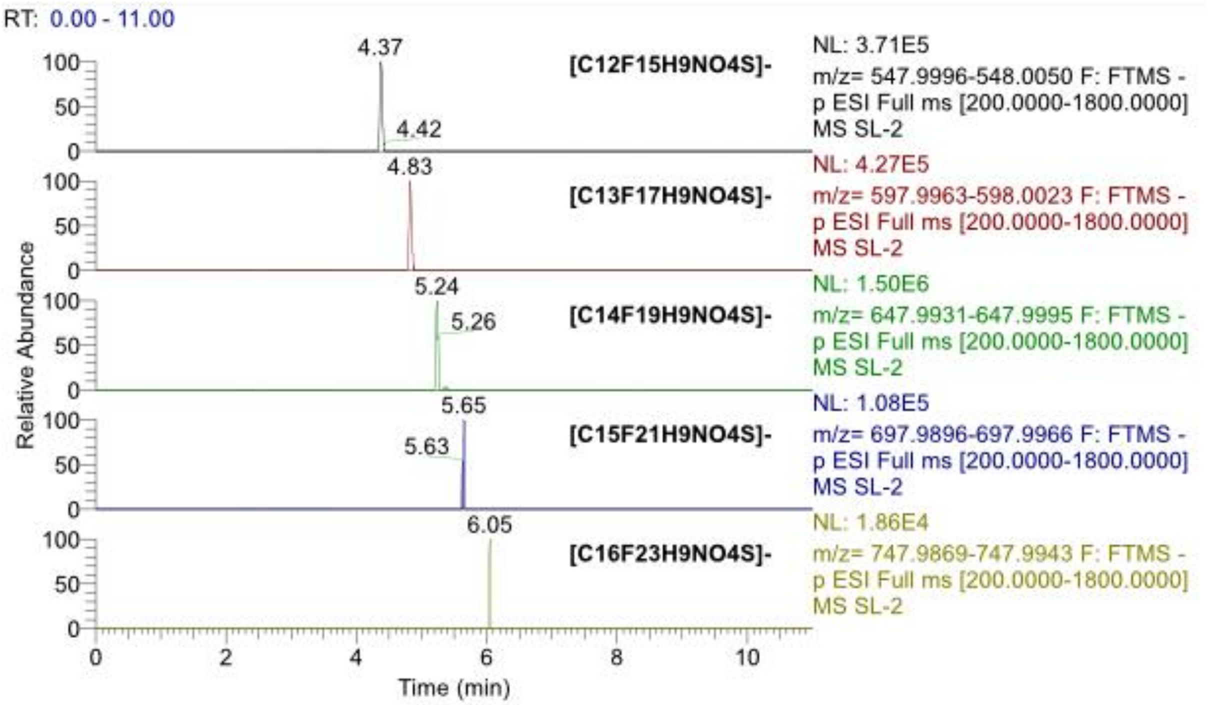

Figure S15: Extracted Ion Chromatograms of unknown homologue $C_nF_{2n-9}H_9NO_4SH$ class in liver sample of Swedish dolphin (SD2).

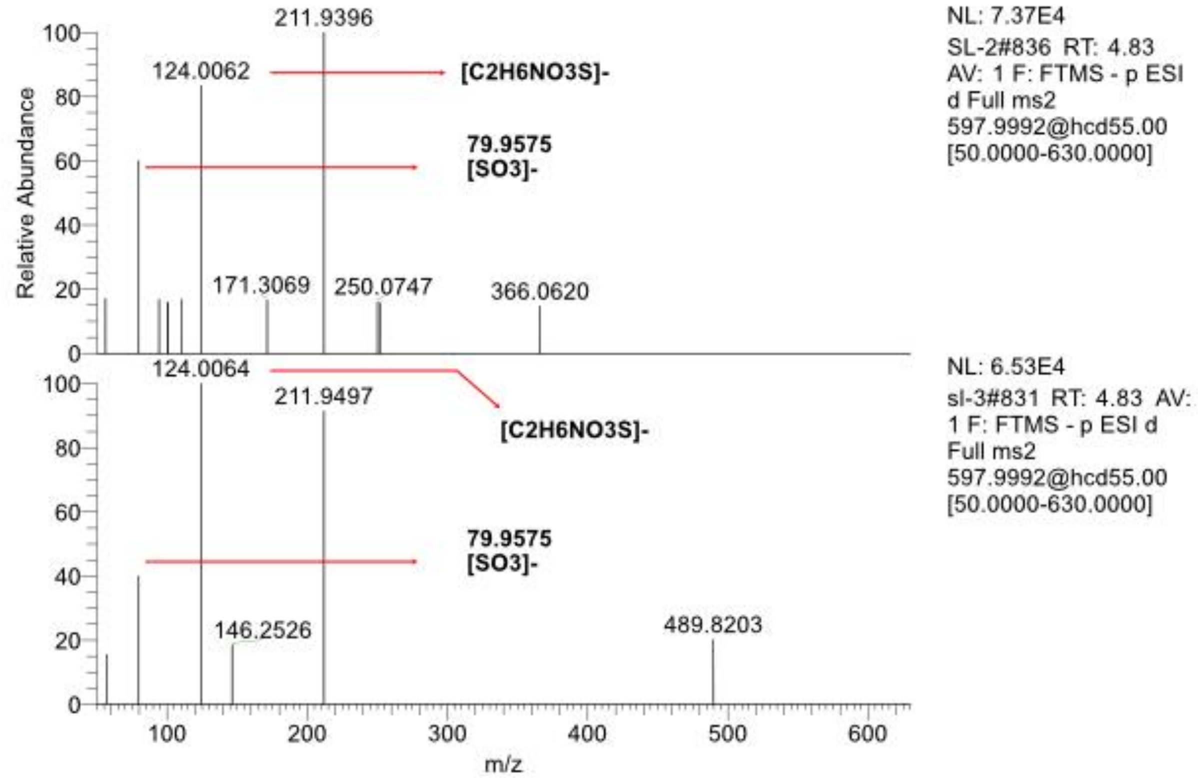

Figure S16: MS^2 of [C13F17H9NO4S]- in liver samples of SD2 and SD3.

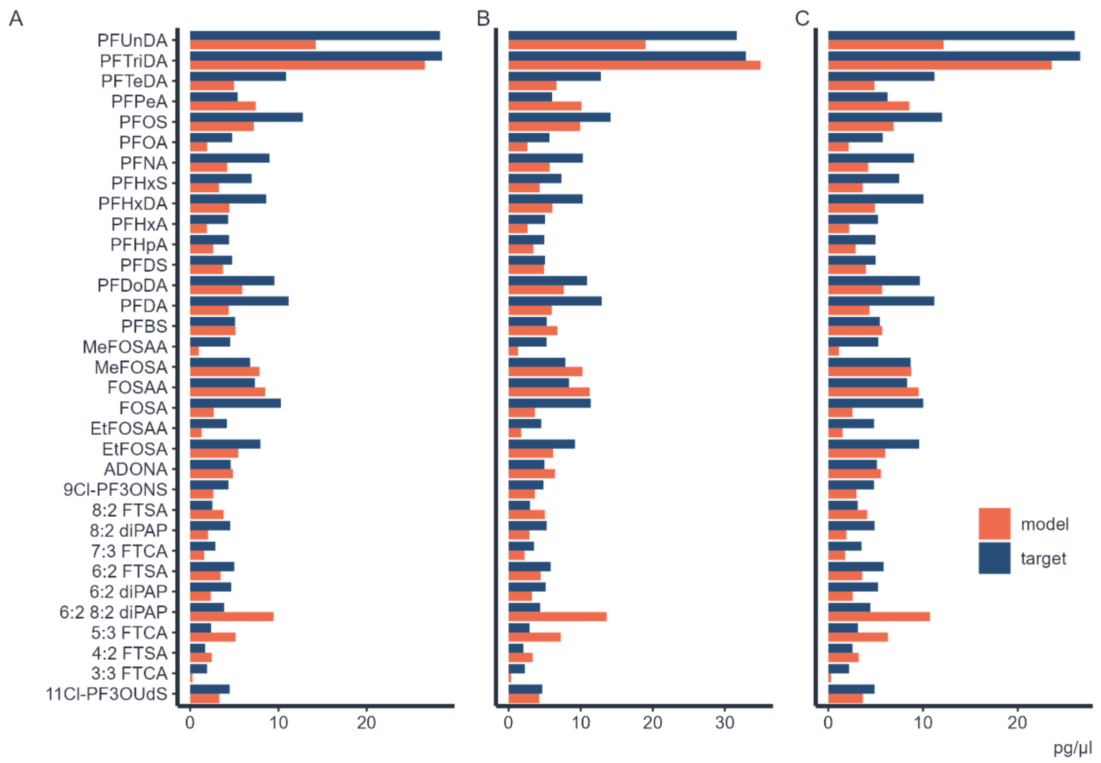

Figure S17: Target and model quantification in three spiked liver samples. Concentrations in pg/µl in final extract.

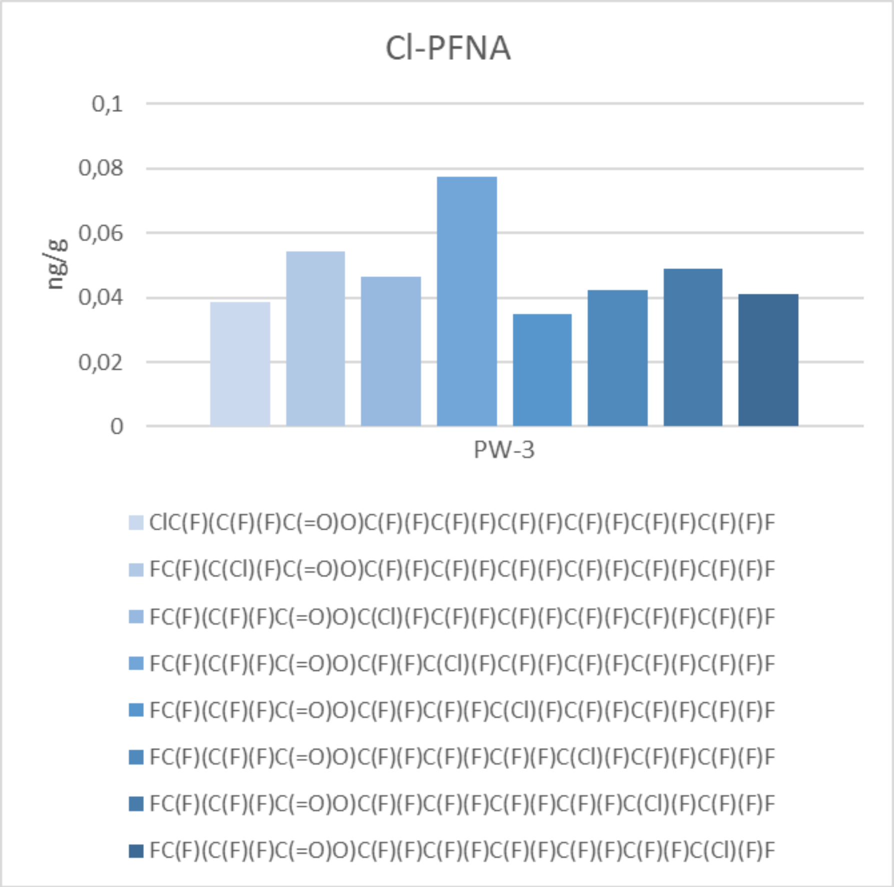

Figure S18: Predicted concentration in pg/ul for different isomers of Cl-PFNA, chlorine position from closest to furthest from carboxyl functional group (left to right). Even though there is variability, predicted concentrations stay in the same order of magnitude.

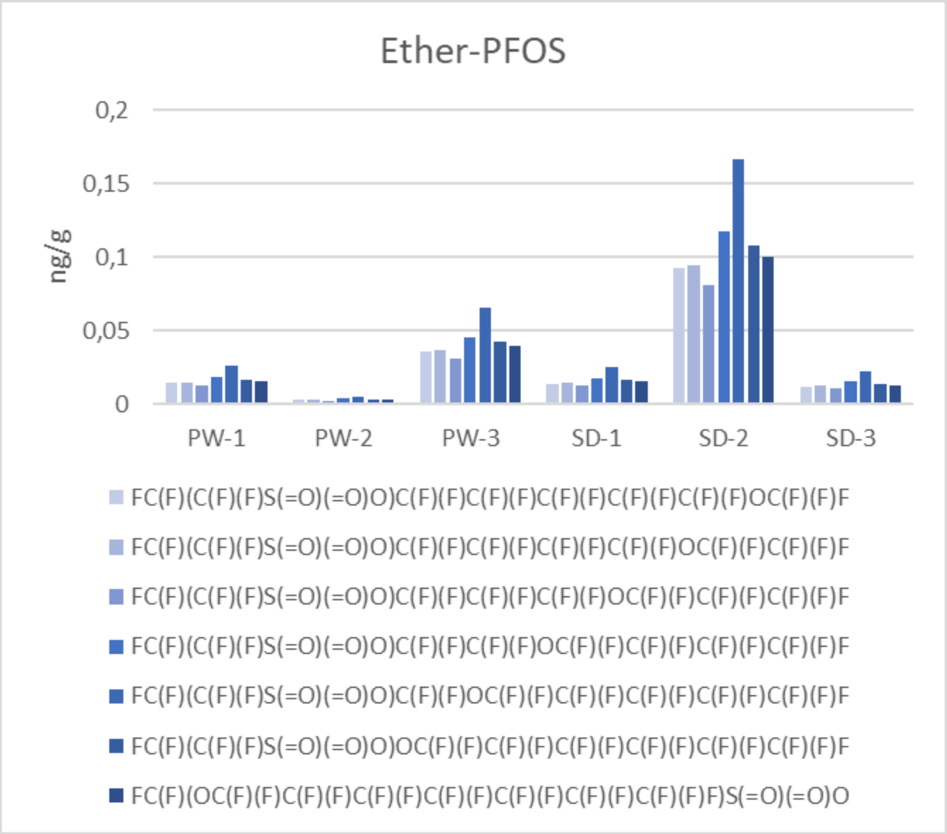

Figure S19: Predicted concentration in pg/ul for different isomers of ether-PFOS, ether position from furthest to closest from sulfonic acid functional group (left to right). Even though there is variability, predicted concentrations stay in the same order of magnitude.

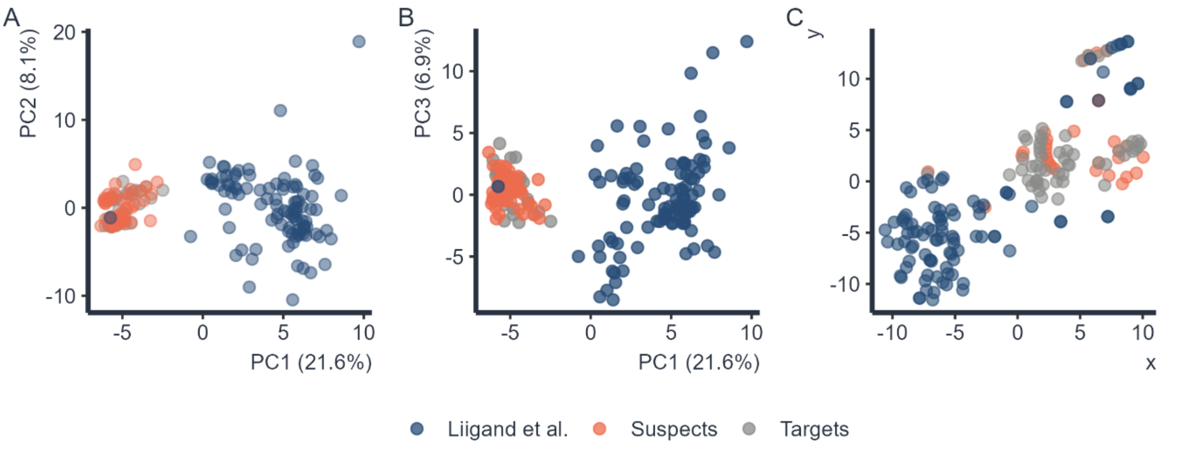

Figure S20: Model application domain was visually assessed with principal component analysis (PCA) and *t*-distributed stochastic neighbour embedding (*t*-SNE) analysis to ensure that the suspect chemicals are similar to chemicals used in training. The results for targets, suspects and non-PFAS included in Liigand et al. of A) first and second principal component, B) first and third principal component and C) t-SNE analysis confirmed that the targets added to the training set overlapped with the suspects and therefore, the ionization efficiency prediction model is appicable on suspect PFAS.
